# Against All Odds: Trehalose-6-Phosphate Synthase and Trehalase Genes in the Bdelloid Rotifer *Adineta vaga* Were Acquired by Horizontal Gene Transfer and Are Upregulated during Desiccation

**DOI:** 10.1371/journal.pone.0131313

**Published:** 2015-07-10

**Authors:** Boris Hespeels, Xiang Li, Jean-François Flot, Lise-Marie Pigneur, Jeremy Malaisse, Corinne Da Silva, Karine Van Doninck

**Affiliations:** 1 LEGE laboratory, URBE, Department of Biology, University of Namur, Namur, Belgium; 2 Department of Genetics, Evolution and Environment, University College London, London, United Kingdom; 3 Research Unit for Molecular Physiology, Cell and Tissue Laboratory, Namur, Belgium; 4 CEA-Institut de Génomique, GENOSCOPE, Centre National de Séquençage, Evry, France; 5 naXys, Namur Center for Complex Systems, University of Namur, Namur, Belgium; University of Nebraska-Lincoln, UNITED STATES

## Abstract

The disaccharide sugar trehalose is essential for desiccation resistance in most metazoans that survive dryness; however, neither trehalose nor the enzymes involved in its metabolism have ever been detected in bdelloid rotifers despite their extreme resistance to desiccation. Here we screened the genome of the bdelloid rotifer *Adineta vaga* for genes involved in trehalose metabolism. We discovered a total of four putative trehalose-6-phosphate synthase (TPS) and seven putative trehalase (TRE) gene copies in the genome of this ameiotic organism; however, no trehalose-6-phosphate phosphatase (TPP) gene or domain was detected. The four TPS copies of *A*. *vaga* appear more closely related to plant and fungi proteins, as well as to some protists, whereas the seven TRE copies fall in bacterial clades. Therefore, *A*. *vaga* likely acquired its trehalose biosynthesis and hydrolysis genes by horizontal gene transfers. Nearly all residues important for substrate binding in the predicted TPS domains are highly conserved, supporting the hypothesis that several copies of the genes might be functional. Besides, RNAseq library screening showed that trehalase genes were highly expressed compared to TPS genes, explaining probably why trehalose had not been detected in previous studies of bdelloids. A strong overexpression of their TPS genes was observed when bdelloids enter desiccation, suggesting a possible signaling role of trehalose-6-phosphate or trehalose in this process.

## Introduction

Rotifers are microscopic invertebrates characterized by a ciliated head structure and a jaw-like grinding organ, the mastax. Within the phylum Rotifera, bdelloids are of particular interest because of their survival and diversification for tens of million years without males, reproducing asexually [1,2]. Recently, our team revealed a particular genome structure in the bdelloid lineage *Adineta vaga* confirming this ameiotic evolution: the allelic regions were rearranged and sometimes found on the same chromosome preventing pairing of homologues chromosomes and proper segregation of alleles. Our genomic results and a recent transcriptomic study by Boschetti et al. [3] on the closely related bdelloid species *Adineta ricciae* also revealed that around 8% of the genes in the genome are of apparent non-metazoan origin and were therefore probably acquired horizontally. In *A*. *ricciae*, these genes of foreign origin appear to be expressed on a scale unprecedented in animals. Moreover, there is evidence for ancient HGTs in the genus *Adineta* when comparing both species [4] suggesting that HGTs have contributed significantly to the adaptation of bdelloid rotifers and may represent an advantage during their ameiotic evolution.

The 400 described morphospecies of bdelloid rotifers are ubiquitous inhabitants of nearly all freshwater habitats on Earth, with a preference for limno-terrestial environments such as mosses, lichens and temporary freshwater pools [5]. Their ability to thrive in such habitats lies in their extreme resistance to desiccation at any stage of their life cycle. When habitats dry out, bdelloids enter a state of suspended life in the form of a “tun” (when their body becomes ovoid and contracted), then regain activity when water becomes available. This phenomenon, known as “anhydrobiosis” [6], was discovered in bdelloids over 300 years ago by Van Leeuwenhoek [7]. Anhydrobiosis is not common among metazoans and is often restricted to particular developmental stages such as the resting eggs of the monogonont rotifers [8] and *Daphnia* [9], the cysts of some crustaceans such as *Artemia* [10], and the larvae of a few insect species, including chironomids [11]. However, there are three well-known invertebrate phyla able to withstand desiccation at any stage in their life-cycle, including the adult form: tardigrades, nematodes and bdelloid rotifers [12]. These taxa represent unique model systems to investigate the desiccation phenomenon.

Desiccation survival involves complex changes and adaptations at morphological, physiological and molecular levels that either protect cellular components from desiccation-induced damage or promote their repair following rehydration. A number of candidate genes involved in desiccation resistance have been identified by genomic, transcriptomic and proteomic analyses in several species. There appears to be a set of molecular adaptations common to all “anhydro-organisms”, which has led Potts et al. [13] to coin the term “desiccome” to describe those “universal” molecules involved in desiccation tolerance. One of the critical components of the desiccome are non-reducing disaccharides found to accumulate during desiccation, such as trehalose (in bacteria, fungi, some resurrection plants and metazoans) and sucrose (in most plants, pollen and seeds) [14–16]. These sugars have been proposed to play a role in osmotic adjustment, to stabilize biomolecules and membranes, and to act as a replacement for water [17,18].

In Archaebacteria an accumulation of trehalose occurs in response to stress suggesting that the protective role of trehalose during cell dehydration might be an ancient adaptation that is evolutionary preserved [19,20]. Accumulation of high levels of trehalose was also observed in desiccated cysts of *Artemia fransiscana* and the desiccation tolerant chironomid larvae up to 15% and 20% respectively of dry body mass [10,21]. Accumulation of trehalose or overexpression of TPS genes upon desiccation was also reported in numerous desiccation-resistant nematodes [22,23]. In tardigrades, trehalose was found in all investigated species but the level was close to detection limit in the species *Milnesium tardigradum* and there appears to be divergence in the responses to desiccation, suggesting that trehalose is not be the unique key molecule for desiccation resistance in these organisms [24,25].

Despite the presence of trehalose anabolytic and catabolytic enzymes in the whole plant kingdom, high amounts of trehalose (up to 12% of dry weight) were only detected in few desiccation-tolerant plants [20,26]. Surprisingly, attempts to improve drought tolerance by engineering genetically modified plants accumulating trehalose resulted in plants that were less tolerant to drought than wild-type strains [27]. There are therefore evidences that trehalose produced by plants does not primarily act as a bioprotectant. Instead, sucrose is hypothesized to take over the role of trehalose as a protective sugar during desiccation in plants [26,28]. Even in a highly desiccation-tolerant plant such as *Selaginella lepidophylla*, a recent publication suggested that the high level of trehalose detected is synthetized by plant endophytes and not by the plant itself [20]. The widespread occurrence and evolutionary conservation of genes involved in trehalose metabolism in the plant kingdom suggests their possible implication in something else than mere desiccation or drought resistance by trehalose accumulation. Indeed, in the last decade it has been highlighted repeatedly that the important roles of TPS and trehalose-6-phosphate are in regulating plant metabolism, growth, development and abiotic stress response [29–32].

Among the five biosynthetic pathways for trehalose recognized to date, the TPS/TPP pathway is the most common: it is found in the three domains of life and is the only pathway utilized by plants and metazoans (summarized in [33]). In this pathway, the synthesis of trehalose from glucose is catalyzed by the enzymes TPS and trehalose phosphatase (TPP) (Figure A in S1 File). The degradation of trehalose, on the other hand, most commonly involves the enzyme trehalase (TRE), which catalyzes the breakdown of trehalose into two D-glucose molecules (Figure A in S1 File) [33].

Surprisingly, disaccharides such as trehalose have never been detected in the bdelloid rotifers while most of the species appear desiccation resistant. Carbohydrate analysis by gas chromatography (GC) of samples extracted from desiccated *Philodina roseola*, *A*. *vaga* and *Macrotrachela quadricornifera* yielded no evidence of trehalose or any other disaccharide, and all attempts to amplify bdelloid TPS genes failed [34–36]. A recent analysis of a cDNA library of the bdelloid rotifer *Adineta ricciae* enriched for genes upregulated following dehydration for 24h at 98% relative humidity (RH) did not detect any genes involved in the biosynthesis of non-reducing disaccharides such as trehalose [37]. By contrast, in the resting eggs of the facultative sexual rotifers of the class Monogononta, the sister clade of bdelloids, trehalose was detected using GC [38]. Furthermore, EST sequencing revealed that sexual as well as amictic females of the monogonont species *Brachionus plicatilis* expressed TPS [8,38]. However, in contrast to the desiccated cysts of *Artemia fransiscana*, a low amount of trehalose (0.35% trehalose/dry weight) was detected in desiccated resting eggs of monogononts suggesting that trehalose may be involved in osmotic regulation in these organisms rather than in resistance to desiccation [36]. It is nevertheless surprising that the bdelloid rotifers lack trehalose metabolism while this sugar is present, even at low concentrations, in all desiccation resistant metazoans.

The recently sequenced genome of *A*. *vaga* comprises the most diverse repertoire of carbohydrate-active enzymes (CAZymes) reported among metazoans so far, including 623 glycoside hydrolases (GHs, involved in the hydrolysis of sugar bonds), and 412 glycosyltransferases (GTs, responsible for building sugar bonds) [3]. Inspired by this rich repertoire, the genome of *A*. *vaga* was screened here for candidate genes involved in trehalose biosynthesis and degradation and the phylogenetic origin of the TPS and TRE enzymes of bdelloid rotifers was verified. Moreover, cDNA libraries and quantitative real-time PCRs (qPCRs) were conducted at different time points before, during and after desiccation of *A*. *vaga* to determine the expression profile of the detected genes involved in trehalose metabolism. While we did not study the activity of the detected trehalose enzymes, our results start to lift the veil on the origin of the trehalose genes of *A*. *vaga* and their metabolism.

## Materials and Methods

### Bdelloid rotifer cultures

Experiments were performed using isogenic *A*. *vaga* clones issued from a single individual from the laboratory of Matthew Meselson at Harvard University [39]. The cultures were maintained hydrated at 16°C in 150 × 20 mm Petri dishes supplemented with natural spring water (Spa) and fed with *Escherichia coli* MG1655.

### Annotation of candidate genes and sequence divergence calculations

Representative sequences of proteins involved in trehalose biosynthesis and hydrolysis pathways in eubacteria, archaea, plants, fungi and animals (Figure A in S1 File)were downloaded from the NCBI database and aligned against the genome of *A*. *vaga* using TBLASTN [40] as implemented in BioEdit [41] with a E-value threshold of 10^−10^ (as recommended by the program MCScanX to detect homologues within and between genomes [42]). Intron-exon boundaries were identified by comparison with homologous amino acid sequences using GENEWISE2 [43] or, in a few cases, were identified manually based on the GT/AG rules for intron boundaries [44]. Translated CDS were used as queries for BLASTP [45] searches against the CAZyme repertoire found in the *A*. *vaga* genome [39] and the nr GenBank database. For each putative protein, the alien index (AI) [46] was determined as in [39]. Genes present on the genomic contigs of *A*. *vaga* were predicted according to the automated annotation of *A*. *vaga* [39] supplemented with manual curation and functional annotation as in Hur et al. [47].

The well-characterized sequence of *E*. *coli* TPS (OtsA), the 3D structure of which had been experimentally determined [48,49], was aligned with the TPS sequences found in *A*. *vaga* in order to check for conservation of amino acid residues involved in the binding of glucose 6-phosphate (acceptor) and UDP-glucose (donor). The alignment of the proteins was performed using MUSCLE [50] and manually edited with MEGA 6.06 [51] to remove non-conserved regions. Based on the previous protein alignment, a 3D-structure modeling of *AvTpsA* was performed using SWISS-MODEL (in Alignment Mode) [52–54] based on the known tridimensional structure of the OtsA chain B homotetramer in complex with G6P and UDP (1GZ5 [48]). A 3D alignment with the original 3D structure of OtsA was obtained using Pymol v1.3. In order to look for 3D conservation of active residues, an additional 3D structure of TPS1 was modeled using Phyre2 [55]. This second model was designed based on the 3D structure of OtsA chain A in complex with UDP-2-deoxy-2-fluoroglucose [49].

The sequence divergence between the identified candidate genes of *A*. *vaga* was calculated at the gene and protein sequence level using Geneious 5.4.6 (Biomatters).

### Sequencing *A*. *vaga* candidate *tps* and *tre* genes

Two overlapping sets of PCR primers for each *tps* gene copy and one set of primer for each *tre* gene copy identified in the genome of *A*. *vaga* were designed using Geneious 5.4.6 and BatchPrimer3 v1.0. Each 25 μL PCR contained 1X GoTaq reaction buffer (1.5 mM MgCl_2_), 0.2 mM of each dNTP, 0.5 μM of each primer, 0.5 U of GoTaq DNA Polymerase (Promega), and 1 μL (ca. 10–200 ng) of genomic DNA. The amplification profile included an initial denaturation step at 94°C for 4 min, followed by 30 cycles of 45 s denaturation at 94°C, 45 s annealing at the appropriate temperature for each primer pair, and 50 s elongation at 72°C; and a final elongation step of 10 min at 72°C. The amplified fragments were Sanger-sequenced in both directions at Genoscreen (Lille, France) to confirm the sequences of the identified *A*. *vaga tps* and *tre* genes. Primers used for amplifying the trehalose-6-phosphate synthase (TPS) and trehalase (TRE) genes are listed in Table A in S2 File.

### Phylogenetic analysis

Since HGT is apparent in bdelloid rotifers, the phylogenetic trees were build based on an exhaustive search of TPS and TRE genes found in the different domains of life. The selection of all TPS genes given in Avonce et al. [33] were supplemented with metazoan sequences from the Uniprot database. Moreover, sequences homologous to *A*. *vaga* genes were selected from the best BLASTP hits against the non-redundant protein database of NCBI. For the trehalase phylogenies, a sample of homologous TRE genes was selected from the Swiss-Prot database using BLASTP, then enriched with the best BLASTP hits of *A*. *vaga* genes against the non-redundant protein database of NCBI. Unpublished monogonont trehalase sequences were provided by Dr. David Mark Welch (MBL, Woods Hole).

The complete list of sequences used in our study is provided in Table B in S2 File. All TPS and TRE amino acid sequences were aligned using MUSCLE [50] in MEGA 6.06 [51]. The alignments were checked manually, then the best-fitting evolutionary model for each alignment was evaluated in PROTTEST 3.4 [56] using the Bayesian Information Criterion [57] and the Akaike Information Criterion [58] (and concurred in selecting the WAG+I+G+F and LG+I+G models for the TPS and TRE phylogenies, respectively).

Neighbor joining trees were constructed in MEGA 6.06 [51] using the JTT model and 1000 bootstraps. Maximum-likelihood trees were constructed in raxml GUI v1.3 [59,60] with 1000 rapid bootstraps according to the same evolutionary model as PROTTEST results. A Bayesian phylogeny was inferred using MrBayes v3.1.1 [61] as implemented in Topali v2.5 [62]. Two runs of 2.5 and 3 million generations were conducted for TPS and TRE respectively, sampling trees every 10 generation and discarding the first 25% as burn-in. The PSRF (Potential scale reduction factor) was in both case equal to 1, meaning that excellent convergence had been achieved. We also used the probability-consensus pruning method (under maximum likelihood) implemented in MetaPIGA v3.1 [63]. The program selected the best-fitting protein substitution model (WAG+G+F and GTR20+G for TPS and TRE respectively) implementing the Bayesian Information Criterion. To estimate the posterior probability distribution of possible trees, replicated metaGA searches were performed and stopped when a series of mean relative error values among 10 consecutive consensus trees remained below 5% (between 100–10,000 replicates). Trees were edited using FigTree v1.4.0 (http://tree.bio.ed.ac.uk/software/figtree/).

### Desiccation assays

Bdelloid rotifers of *A*. *vaga* were desiccated following the protocol published in [64]: briefly, healthy *A*. *vaga* cultures were washed with filtered Spa water, individuals were collected using a cell scraper, transferred to a 50-ml Falcon tube and centrifuged. Concentrated bdelloid individuals were transferred with 1,2 ml supernatant to the center of Petri dishes containing 3% LMP agarose (Invitrogen Carlsbad). Plates with similar amount of bdelloids were placed in a climatic chamber (WEKK 0028) and submitted to the following desiccation protocol: first a linear decrease of relative humidity from 70% to 55% for 17 h, then a linear decrease of relative humidity from 55% to 41% for 1 h, and maintenance of relative humidity at 41% during the 14 days of desiccation. *A*. *vaga* individuals were rehydrated by adding 30 mL filtered Spa water to each plate. During all the experiment, temperature was kept at 23°C. RNA for qPCR analysis and/or cDNA preparation was extracted at six different time points before, during or after desiccation (Figure B in S1 File).

### RNA extraction and cDNA library construction

Total RNAs were extracted using the RNAqueous-4PCR Kit (Ambion, Austin) at the six time points (Figure B in S1 File). Total RNA was enriched in mRNA based on its polyA tail, chemically fragmented and converted into single-stranded cDNA using random hexamer priming, generating double-stranded cDNA. Next, paired-end libraries were prepared following the Illumina 222s protocol (Illumina DNA sample kit): briefly, fragments were end-repaired, 3’-adenylated, and ligated with Illumina adapters. DNA fragments ranging in size from 300 to 600bp (including the adapters) were PCR-amplified using adapter-specific primers. Libraries were purified then quantified using a Qubit Fluorometer (Life technologies), and library profiles were evaluated using an Agilent 2100 bioanalyzer. A paired-end flow cell of 101-bp reads was sequenced for each library on an Illumina HiSeq2000 platform. Raw data were normalized per kilobase of gene and per billion of mapped reads (RPKB) to allow comparison between conditions. The cDNA reads were mapped against the putative open reading frames (ORFs) of the identified TPS and TRE genes using Gaze v2 [65] to quantify their expression at the different time points.

### Quantitative PCR

The expression levels of the TPS and TRE genes at each time point (Figure B in S1 File) were confirmed by conducting qPCRs. RNA extraction for the experiment followed the protocol detailed above. Each cDNA synthesis reaction was performed using 390 ng of total RNA, 200 U of M-MLV Reverse Transcriptase (Promega), 80 μM dNTP mix (Promega) and 15 ng/μL random hexamers (Invitrogen) in 20 μL of 1X M-MLV reaction buffer (Promega M531A) for1 h at 42°C, then exposed for 5 min at 95°C and kept on ice. FastStart Universal SYBR Green Master (Rox) (Roche, Basel, Switzerland) was used for qPCR. The oligonucleotide primer sequences (300 nmol/L; IDT) for TPS and TRE genes are listed in Table C in S2 File. All amplification products were checked using BLAST searches against the published genome of *A*. *vaga*, gel visualization, dissociation curves and sequencing.

After investigating the stability of the expression of eight possible housekeeping genes (18S, 28S, 53A, L40, L32, α tubulin, β tubulin and actin) across 12 Illumina RNA-seq libraries obtained at different time points (see [39]) the L40 ribosomal subunit was chosen for the qPCR analyses because it had the most stable expression pattern (stdev/average = 0.54). Each time point was investigated in triplicate (to account for environmental variation) and each qPCR reaction was performed in duplicate (to account for experimental variation). Relative quantifications were performed following the ∆∆Ct method [66] supplemented by analyses of variance (ANOVA1).

## Results

### Identification of the *Adineta vaga* genes involved in trehalose metabolism

#### The trehalose biosynthesis pathway

**Fig 1:**
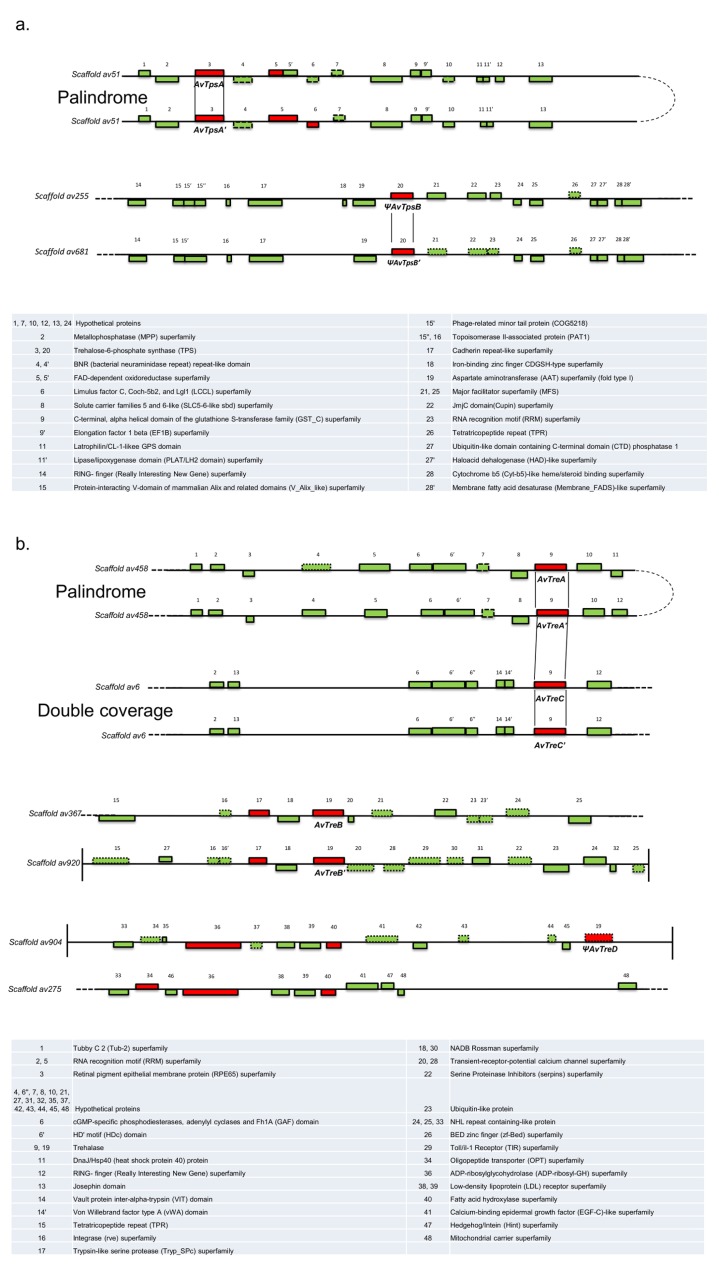
Annotation of the scaffolds containing (a) TPS and (b) TRE genes in *Adineta vaga*. Color-filled rectangles represent genes, numbers refer to the list at the bottom of the figure. Genes were colored in green or red if AI<45 or AI≥45, respectively. The genes annotated automatically in the *A*. *vaga* genome are surrounded with continuous lines, whereas discontinued lines surround additional genes detected during manual curation. The gene orientation is indicated by the position above (forward direction) or below (reverse direction) the line.

The TBLASTN search results for *A*. *vaga* homologues of trehalose phosphate synthase (TPS) from a wide range of species (bacteria, fungi, plants and metazoans) yielded significant E-values (< 10^−10^) for four predicted genes belonging to three different scaffolds (av51, av255 and av681) in the *A*. *vaga* genomic dataset. Indeed, *A*. *vaga* was previously reported to be an ancient degenerate tetraploid [39], hence finding more than two copies of a given gene was not surprising. Two TPS genes were found on a palindromic, highly colinear allelic region [39] of scaffold av51, and were annotated *AvTpsA* and *AvTpsA’* (Fig 1A). The surrounding region of the TPS copies on scaffolds av255 and av681 were also highly colinear and therefore probably allelic [39]: these two copies were annotated as *AvTpsB* and *AvTpsB’* (Fig 1A). One copy (*AvTpsB*) was characterized by the insertion of an A at position 223 (in comparison with *AvTpsB’*) generating a stop codon in the translated protein. In *AvTpsB’*, the substitution of a C by a T at position 766 also resulted in a stop codon and a truncated protein sequence. We scrutinized the corresponding cDNA sequencing dataset and found no evidence for RNA editing (see below), suggesting that these two copies might be pseudogenes and we therefore prefixed their names with the Greek letter Ψ (Fig 1A). In downstream phylogenetic analyses, we restored both genes to their presumable full length by deleting the A in position 223 of Ψ*AvTpsB* and by replacing the T in position 766 of Ψ*AvTpsB’* by a C.

The putative allelic relationships between the copies, inferred from colinearity, were evaluated by calculating the pairwise sequence divergence: *AvTpsA* and *AvTpsA’* were nearly identical and shared 98.2% identity at the nucleotide level and 98.3% at the protein level whereas Ψ*AvTpsB* and Ψ*AvTpsB’* shared 96.9% identity at the nucleotide level and 94.5% at the protein level. The two pairs (A and B) containing those genes were 71.3% identical at the nucleotide level and 57.0% identical at the protein level. Although synteny was well conserved within each pair of TPS genes, no synteny was detected between the two pairs except at the *Tps* position (Fig 1A), suggesting that the four contigs containing the TPS genes of *A*. *vaga* do not form a quartet of ohnologues such as reported previously in *A*. *vaga* for hsp82 or histone H3 [47,67]. All the four *Tps* gene copies had been previously annotated in the repertoire of CAZymes of the *A*. *vaga* genome [39].

Within the *A*. *vaga* genomic dataset no significant hit to the trehalose phosphate phosphatase (TPP) gene present in bacteria, fungi, plants and metazoans (Figure A in S1 File) was detected. The TPP catalytic site of the gene is characterized by three short, highly conserved motifs found in archaea, eubacteria, plants and opisthokonts [33,68], and it has been suggested that TPP domains became fused to the TPS protein in some bacteria and archaea as well as in most eukaryotes [33,69,70]. A close examination of the C-terminal regions of the four predicted TPS proteins of *A*. *vaga* did however not reveal any putative TPP domain.

**Fig 2:**
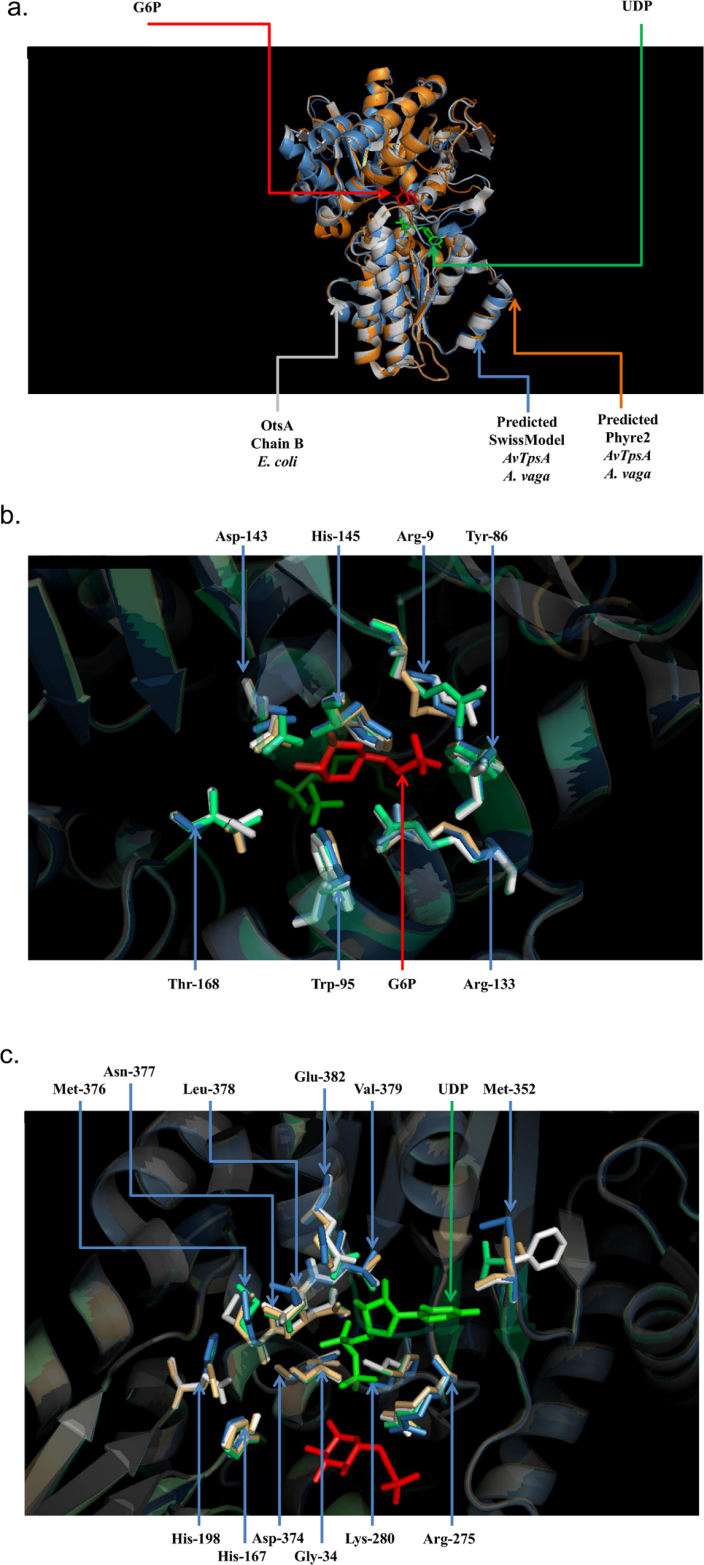
(a.) 3D alignment of the OtsA chain B crystal structure obtained for *E. coli* (white) with the two protein structures of TPS1 modeled using SWISS-MODEL (light blue) and PHYRE2 (orange). UDP (green) and G6P (red) are localized as observed in the OtsA crystal structure. (b.) Close-up on the conserved sites involved in the binding of G6P (red). The residues of the *E*. *coli* OtsA are shown in white and those of the *AvTpsA* predicted using SWISS-MODEL in light blue. SwissModel TPS1 *A*. *thaliana* (light green) and SwissModel predicted TPS1 *P*. *brassicacea* (gold) are also displayed in order to compare their topologies. (c.) Close-up on the conserved sites related to the binding of UPD (green). The residues are labeled based on the 3D structure of *AvTpsA* predicted using SWISS-MODEL.

In order to check whether the amino acid residues important for UDP and G6P binding were conserved in the *A*. *vaga* TPS proteins, their sequences were aligned with the OtsA sequence of *Escherichia coli* and with the TPS sequences of *Arabidopsis thaliana* and *Plasmodiophora brassicae* (respectively *AtTPS1* and *PbTPS1* [71]) that are within a same clade as *A*. *vaga* in our TPS phylogenies (see below). Both *AtTPS1* and *PbTPS* are known to be fully functional. Alignment of the four predicted TPS proteins of *A*. *vaga* with the OtsA protein sequence of *E*. *coli* revealed that 16 out of 19 residues important for UDP and G6P binding were conserved in *AvTpsA* and *AvTpsA’*, whereas 15 and 13 out of 19 of these important residues were conserved in Ψ*AvTpsB* and Ψ*AvTpsB’* respectively (Figure C in S1 File). By comparison, 16 out of 19 residues are conserved between *A*. *thaliana* and *E*. *coli* [69] and 17 out of 19 are conserved between *P*. *brassicae* and *E*. *coli*. Interestingly, 18 out of 19 residues were conserved between *A*. *vaga AvTpsA-A’* and *A*. *thaliana* TPS1 and 17 out of 19 with *P*. *brassicae* TPS1 (Figure C in S1 File). The predicted 3D models of *AvTpsA* were close to published structure of the OtsA of *E*. *coli*, with a root-mean-square equal to 0.166 Å and 0.234 Å for the 3D structures predicted by SwissModel and Phyre2 respectively (Fig 2A and 2B). Comparing the residues involved in UDP (Fig 2B) and G6P binding (Fig 2C) between the crystal structure of OtsA and the predicted protein structure of *AvTpsA*, *AtTPS1* and *PbTPS1* also suggested that the topology of these residues was conserved (despite few amino acid substitutions between the tested species).

No homologues of the genes involved in the TreP, TreT and TreY pathways of trehalose biosynthesis (Figure A in S1 File) were detected in the genome of *A*. *vaga*.

#### The trehalose hydrolysis pathway

The TBLASTN alignments of the TRE proteins from a wide range of species (bacteria, fungi, plants and metazoa) against the *A*. *vaga* genome yielded six significant hits (E-values < 10^−10^) to five different scaffolds (av458, av6, av367, av920, av904) (Fig 1B and Table 1). Two TRE genes were found on scaffold av458 in a palindromic, highly colinear allelic region [3], and were therefore annotated *AvTreA* and *AvTreA’*. The regions surrounding two other TRE copies on scaffold av367 and av920 were highly colinear and therefore likely allelic [39]: these two allelic copies were annotated as *AvTreB* and *AvTreB’*. The TRE gene found on scaffold av6 and the adjacent genes exhibited a coverage twice the genome average suggesting that two allelic TRE copies had been fused: we labeled these two copies *AvTreC* and *AvTreC’*. An additional copy that had not been annotated in the published *A*. *vaga* genome was found in the terminal region of scaffold av904 and labeled *AvTreD*. The genomic region homologous to scaffold av904, scaffold av275, did not contain any detectable trehalase gene copy (Fig 1B), making a total of seven TRE gene copies in the published *A*. *vaga* genome. Since *AvTreD* had a stop codon located at the 886–888 bp of its coding region (and its cDNA sequence did not reveal evidence for RNA editing), we considered it to be a pseudogene and therefore prefixed its name with the Greek letter Ψ. The putative allelic relations between TRE copies were confirmed by analyzing the pairwise divergences between their sequences (see Table D in S2 File). In the regions surrounding the TRE genes on scaffolds av458 and av6, synteny was well preserved (Fig 1B), suggesting that *AvTreA-A'* and *AvTreC-C’* were ohnologues and form a quartet of four homologous regions (as in Hur et al and Van Doninck et al). The three other copies did not present such organization. The two other existing classes of trehalose hydrolytic enzymes (phosphotrehalase and trehalose phosphorylase), which had never been observed in animals so far, were not detected in the *A*. *vaga* genome either.

**Table 1:** Summary of top BLASTP hits for the trehalose-6-phosphate synthase (TPS) and trehalase (TRE) genes of *Adineta vaga* against GenBank.

Gene ID, name	Scaffold	Nb of introns	GC(%)	AI	Best hit E-value	Metazoan best hit E-value	Best hit, taxonomy	Best hit, description
GSADVT00013141001, *AvTpsA*	Av51	1	33.2	165	1.00E-161	4.00E-90	Eukaryota; Protozoa; Rhizaria; Cercozoa	glycosyltransferase family 20 protein
GSADVT00013173001, *AvTpsA’*	Av51	1	33.5	174	4.00E-162	2.00E-86	Eukaryota; Protozoa; Rhizaria; Cercozoa	glycosyltransferase family 20 protein
GSADVT00043720001, ΨAvTpsB*	Av255	0	31.1	82	1.00E-107	9.00E-72	Eukaryota; Fungi; Chytridiomycota	glycosyltransferase family 20 protein
GSADVT00063692001, ΨAvTpsB’ *	Av681	0	31.2	78	1.00E-102	8.00E-69	Eukaryota; Fungi; Dikarya	glycosyltransferase family 20 protein
GSADVT00056365001, *AvTreA*	Av458	0	31.7	140	1.00E-147	7.00E-87	Bacteria; Bacteroidetes; Sphingobacteriia	alpha, alpha-trehalase
GSADVT00056372001, *AvTreA‘*	Av458	0	31.7	140	1.00E-147	1.00E-86	Bacteria; Bacteroidetes; Sphingobacteria	alpha, alpha-trehalase
GSADVT00051563001, *AvTreB*	Av367	1	27	281	0.00E+00	1.00E-78	Bacteria; Bacteroidetes; Cytophagia	alpha, alpha-trehalase
GSADVT00021696001, *AvTreB‘*	Av920	1	26.9	233	6.00E-180	1.00E-78	Bacteria; Bacteroidetes; Cytophagia	alpha, alpha-trehalase
GSADVT00001947001, *AvTreC-C’*	Av6	0	33.6	139	2.00E-149	4.00E-89	Bacteria; Bacteroidetes; Cytophagia	alpha, alpha-trehalase
N/A, ΨAvTreD*	Av904	1	25	226	1.00E-177	9.00E-80	Bacteria; Bacteroidetes; Cytophagia	alpha, alpha-trehalase

* putative pseudogene: the stop codon in the middle was corrected to predict a full-length protein

### Alien index, introns and GC content

All the TPS and TRE genes identified had an alien index AI>45 (Table 1), an indication of their probable non-metazoan origin. At most one intron was detected in these genes (Table 1), which is significantly fewer than reported for probable core metazoan genes of *A*. *vaga* (AI≤-45, mean intron number 7.9 [39]). Seven out of the 11 TPS and TRE genes had a GC content (%) more than one percent below the genome average (33.3% [39]) (Table 1), another signature of plausible horizontal origin (see Flot et al. 2013).

### Phylogenetic placement

#### Phylogenetic tree of TPS gene sequences

**Fig 3:**
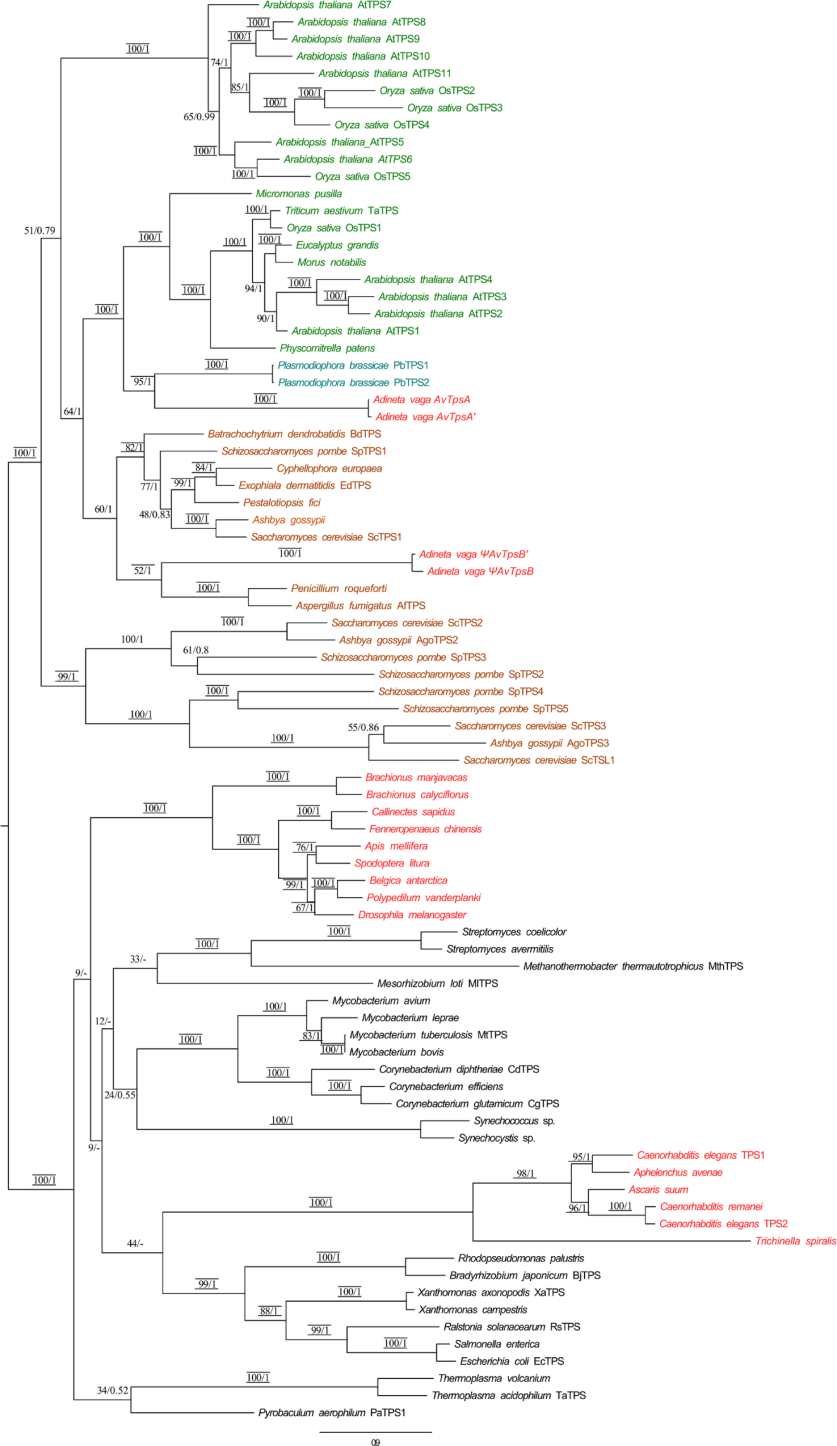
Maximum-likelihood phylogenetic tree based on the amino acid sequences available for trehalose-6 phosphate synthase (TPS) domains. The tree displayed was generated using RaxML with 1000 rapid bootstrap according to the model WAG+I+G+. Numbers above the branches are bootstrap support percentages and posterior probability of 1 obtained with MrBayes (See M&M). When the node was not recovered in the Bayesian tree, the bootstrap value was replaced by “-“. Bootstrap support values above 70% for MetaPIGA and NJ are indicated by lines respectively drawn above and below the ML bootstrap values and posterior probabilities*. Color code: green (plant), red (metazoans), black (bacteria and archeae), cyan (protists) and brown (fungi).

Tree topologies obtained by RaxML, Mr Bayes, Neighbor-Joining and MetaPIGA confirmed that the TPS sequences were grouped into two major branches as previously described by Avonce et al. [33] (Fig 3): the first group contains sequences from fungi, plants and some protists, whereas the second group consists of metazoans and bacteria. The TPS sequences of *A*. *vaga* were part of the clade of plant and fungal TPS proteins (strongly supported by bootstrap branch supports of 100-97-84 for RaxML, NJ, and MetaPIGA respectively, and by a posterior probability of 1 using Mr Bayes). The two *A*. *vaga AvTpsA* and *AvTpsA’* sequences clustered with the protist TPS sequence of *P*. *brassicae* (bootstrap supports of 95-76-62, posterior probability of 1) and formed a sister clade to the plant Class I TPS proteins (bootstrap supports of 100-97-87, posterior probability of 1) confirming their non-metazoan origin. Most of these sequences have biochemically proven TPS enzymatic activity, except *AtTPS2*-*AtTPS4* [71]. The position of Ψ*AvTpsB* and Ψ*AvTpsB’* within the tree was not resolved as the phylogenetic trees were not congruent or branch support was insufficient. In contrast, the TPS sequences of monogonont rotifers (*Brachionus manjavacas* and *Brachionus calyciflorus*) grouped outside the plant and fungal clade and were associated with other metazoans (bootstrap support values 100-100-99, posterior probability 1). As previously described in [33], the nematode sequences did not cluster with those of other metazoans.

### Phylogenetic tree of TRE gene sequences

**Fig 4:**
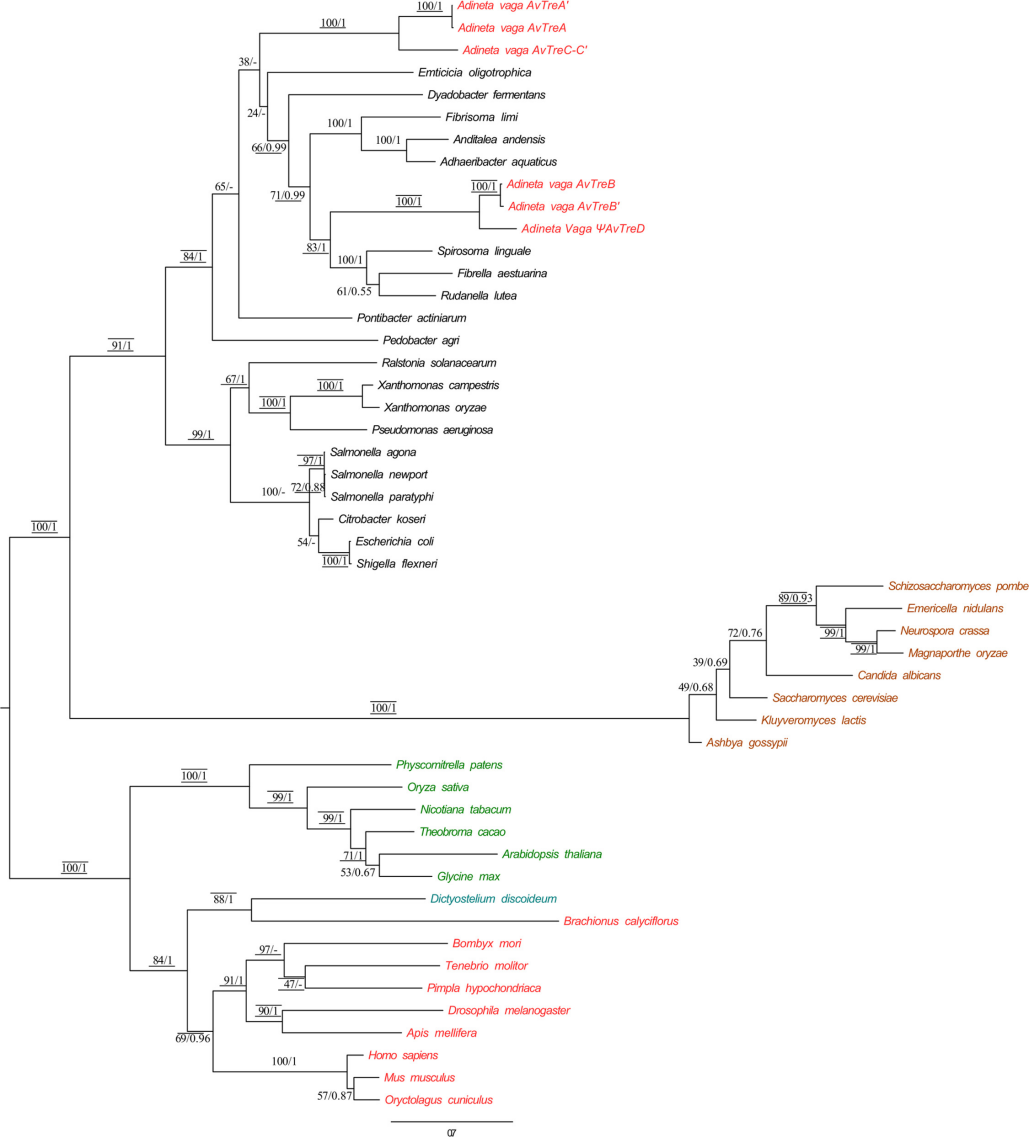
Maximum likelihood phylogenetic tree based on the amino acid sequences available for trehalase (TRE) domains. The tree displayed was generated using RaxML with 1000 rapid bootstrap according to the LG+I+G evolutionary model. Numbers above the branches are bootstrap support percentages and posterior probability of 1 obtained with MrBayes (See M&M). When the node was not found in a Bayesian tree, the bootstrap value was replaced by “-“. Bootstrap support values above 70% for MetaPIGA and NJ are indicated by lines respectively drawn above and below the ML bootstrap values and posterior probabilities. Color code: green (plant), red (metazoans), black (bacteria and archeae), cyan (protists) and brown (fungi).

Unrooted phylogenetic trees (Fig 4) constructed on the basis of the alignment of the amino acid sequences of known or putative TRE predicted the existence of three major clades: one clade of fungal trehalases (bootstrap supports of 100-100-100, posterior probability of 1); one clade of bacterial trehalases (bootstrap supports of 91-100-92, posterior probability of 1); and one clade grouping trehalase sequences of plants, metazoans and some protists (bootstrap supports of 100-100-98, posterior probability of 1). The bacterial clade was further divided into two subclades: one that mostly comprised sequences of the class Bacteroidetes (bootstrap supports of 84-89-85, posterior probability of 1), and a second one that was composed of sequences of the class Proteobacteria (bootstrap supports of 99-100-100, posterior probability of 1). All the seven *A*. *vaga* trehalase sequences fell within the Bacteroidetes clade, confirming their non-metazoan origin. However, it was not possible to resolve more precisely the position of the *A*. *vaga* TRE genes inside Bacteroidetes due to incongruence between trees or poor branch support. In contrast, the TRE sequences of the monogonont rotifer *Brachionus calyciflorus* were closely related to those of plants, metazoans and *Dictyostelium discoideum*. Moreover, a close relation between *D*. *discoideum* and *Brachionus calyciflorus* was observed (bootstrap supports of 88-84-69, posterior probability of 1).

### Expression profiles of the *tps* and *tre* genes

#### RNAseq

To obtain an overview of the expression of the TPS and TRE genes identified in *A*. *vaga*, we analyzed cDNA libraries representing hydrated *A*. *vaga* (control condition), early desiccated *A*. *vaga* (after 37h drying process) and *A*. *vaga* individuals rehydrated since 1.5 hours following 14 days of desiccation (Figure B in S1 File). Mapping the reads obtained from the cDNA libraries against the putative coding sequences of the four *tps* genes identified in *A*. *vaga* revealed few matches to *AvTpsA* and *AvTpsA’* (RPKB<1,000 for each gene in each time point) and no match to Ψ*AvTpsB* and Ψ*AvTpsB’*. For the TRE genes, all the six copies except the putative pseudogene Ψ*AvTreD* were expressed at all time points with a high number of matching ESTs (10^6^−10^8^ RPKB, Figure D in S1 File). At the three time points, the number of reads matching the TRE genes was over thousand times greater than the number of reads matching the TPS genes, indicating a much higher expression level for trehalase compared to trehalose-6-phosphate synthase (Figure D in S1 File).

#### qPCR

**Fig 5:**
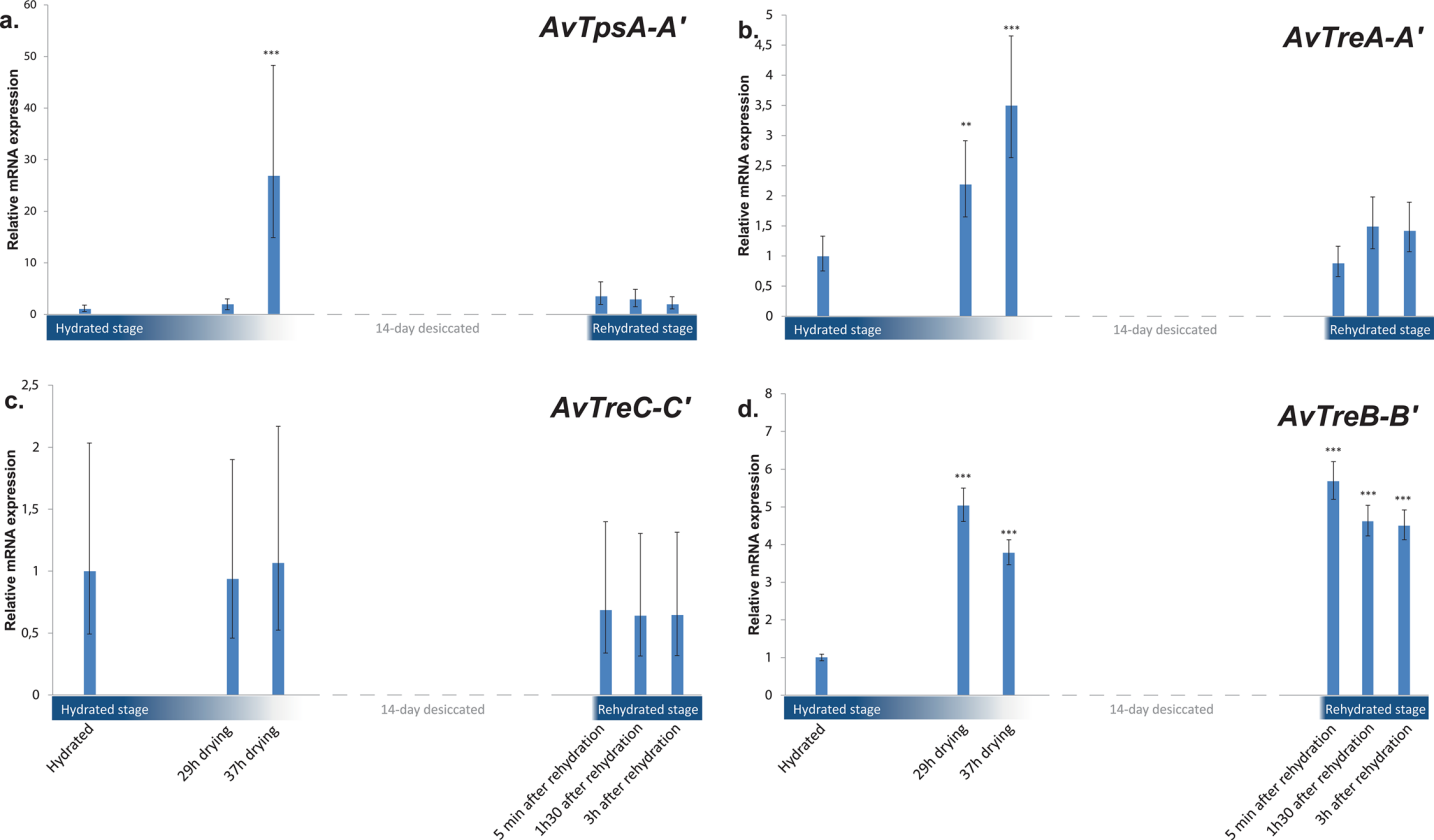
Relative expression of the trehalose-6-phosphate synthase (TPS) and trehalase (TRE) genes in *Adineta vaga* submitted to drying or rehydration: (a.) AvTpsA&A’; (b.) AvTreA&A”; (c.) AvTreC&C’ (d.) AvTreB&B’. The five time points are as follows: (1) hydrated; (2) 28h drying/humidity effect; (3) 37h drying/early desiccated state; (4) 5min rehydration after 14 days of desiccation; (5) 1h30 rehydration after 14 days of desiccation; (6) 3h rehydration after 14 days of desiccation. The expression level of each gene was normalized by reference to the housekeeping gene for the ribosomal protein L40; relative level of expression changes were calculated by reference to that that of the hydrated time point (value = 1). Error bars represent mean value 95% confidence intervals based on three replicates (one-way analysis of variance RM, *P<0.05 ** P<0.01 *** P<0.001).

Quantitative PCRs (qPCRs) were performed on the TPS and TRE genes of *A*. *vaga* to study in detail their expression kinetics during desiccation. We screened five time points during the drying process, desiccated state and rehydration process and compared them with hydrated (control) bdelloids (Figure B in S1 File). We found an increase of *AvTpsA* and *AvTpsA’* mRNA expression levels during drying that reached a peak of 25 fold at 37 h drying (newly desiccated state) in comparison with the control, then decreased during rehydration (Fig 5A). The difference in relative expression is significant at timepoint 37h of drying.

Similar to the expression pattern of *AvTpsA-A’*, the trehalase copies *AvTreA* and *AvTreA’* were upregulated during drying process with a peak at 37 h and a decrease down to control level after rehydration (Fig 5B). At 29h and 37h of drying process the relative expression is significantly higher than the control. In contrast to *AvTreA* and *AvTreA’*, *AvTreC-C’* copies did not show any significant difference between the tested conditions and the control (Fig 5C). Finally, *AvTreB*-*B’* appeared upregulated during both drying and rehydration processes in comparison with the control (Fig 5D).

## Discussion

In contrast to all previous studies reporting the absence of trehalose and its biosynthetic pathway in bdelloid rotifers [34–37], our screening of the published genome of *A*. *vaga* revealed the presence of trehalose-synthesizing (trehalose-6-phosphate synthase) and hydrolyzing (trehalases) genes in the bdelloid rotifer *A*. *vaga*. Some of the copies of both the synthase and hydrolase gene were expressed only at specific time points during the drying-rehydration process (Fig 5.). In addition, the active site residues of the TPS domains were highly conserved in all the four TPS gene copies of *A*. *vaga* (Figure C in S1 File), especially in the *AvTpsA* pair. Moreover the modeled 3D structures of *AvTpsA* suggest that the organization of the active site is also conserved (Fig 2). The genes however appear to be acquired by HGT and as in plants, the trehalose-6-phosphate synthesized by the TPS gene might serve as a signaling molecule when bdelloids become desiccated (see below).

### Evidence for the acquisitions of the *tps* and *tre* genes of *A*. *vaga* by horizontal gene transfer

The four *A*. *vaga* TPS genes have AI>45 and are therefore of probable non-metazoan origin. This was confirmed using different phylogenetic methods: all four *A*. *vaga* TPS sequences fell within the plant-fungal TPS clade (including the protist sequences of *P*. *brassicae*) with strong support (Fig 3). *AvTpsA-A’* formed a sister clade to the clade grouping plant Class I TPS proteins and the protist sequence of *P*. *brassicae*. The TPS genes Ψ*AvTpsB* and Ψ*AvTpsB’* appeared to form a sister group to the plant and fungi Class I/ II TPS clade (including *P*. *brassicae*) but the origin of these *A*. *vaga* TPS domain sequences remains unresolved (Fig 3). The presence of stop codons at two different positions in Ψ*AvTpsB* and Ψ*AvTpsB’* suggests that pseudogenization happened after their acquisition. In the absence of selective pressure because of a plausible redundancy with *AvTpsA-A’*, Ψ*AvTpsB* and Ψ*AvTpsB’* may have diverged considerably from all other TPS genes studied and their position within the plant- fungi clade is therefore unresolved.

The two copies within each pair (*AvTpsA* and *AvTpsA’*, Ψ*AvTpsB* and Ψ*AvTpsB’*) are nearly colinear but *AvTpsA-A’* does not appear to be ohnologous to *AvTpsB-B’*. Therefore, we hypothesize that bdelloid rotifers acquired two different *tps* gene copies (A and B) by two independent horizontal gene transfer events. These two independently acquired *tps* genes may have become duplicated during a gene conversion event or at the time of the whole genome duplication event that is supposed to have occurred early in bdelloid rotifer evolution [5,35], resulting in the two colinear pairs we found. However, we cannot exclude that this duplication occurred in a sexual ancestor of extant bdelloids, in which case, meiotic recombination may have been involved in the transition from one copy to two copies of each of the two genes. Determining whether this acquisition was recent or very ancient among bdelloids will require comparative analysis with other bdelloid species.

The seven *A*. *vaga* trehalase sequences fell within the bacterial TRE clade (Fig 4), and all the trehalase gene copies had AI>45 with one or no intron, indicating that the TRE genes of *A*. *vaga* may have been acquired by horizontal gene transfer from bacteria. The putative horizontal gene transfers involving TRE genes appear relatively ancient, since (1) the trehalase gene copies *AvTreA-A’* and *AvTreC-C’* on scaffolds av458 and av6 are organized in a quartet of four homologous regions (Fig 1B), and (2) the three TRE gene copies *AvTreB*, *AvTreB’* and Ψ*AvTreD* on scaffolds av367, av920 and av904 have acquired one intron each (Table 1).

Horizontal gene transfer appears to be rampant in bdelloid rotifers [4,39,46], and some genes of foreign origin were previously reported to be intact and expressed, as for the TPS and TRE genes in our study. Boschetti et al. [37], studying gene expression in response to desiccation, did not detect expression of any trehalose gene probably because the critical time point of 37h drying (at which the rotifer can be considered as having reached anhydrobiosis as its water content is reduced to about 5% [64]) was not included in their study. The authors however confirmed the existence of a high proportion (8–9%) of foreign genes in the bdelloid genome that appear to be transcribed. Those results suggest that bdelloid rotifers can capture exogenous genes and that these appear to be functional. Horizontal gene transfers in bdelloid rotifers most likely occur during desiccation when membranes are disrupted and DNA is broken and then repaired [64,72,73].

### Why did previous studies fail to detect TPS genes in bdelloid rotifers?

Previous studies using degenerate primers were not able to amplify any TPS fragment in the three bdelloid species tested [34,35]. The degenerate primers used in those studies match poorly the four TPS gene sequences of the present study: the first eight nucleotides of the forward primer were different from the *A*. *vaga* TPS gene sequences, whereas numerous differences were observed between the reverse primer and the *A*. *vaga* TPS gene sequences (Figure E in S1 File). Indeed, Lapinski and Tunnacliffe [34] defined their primers based on an alignment of TPS sequences from bacteria, yeast and metazoans, whereas the TPS genes of *A*. *vaga* are more closely related to plants for *AvTpsA* and *AvTpsA’* and to fungi or plant for Ψ*AvTpsB* and Ψ*AvTpsB’*.

Previous studies also failed to detect TPS gene expression as well as trehalose accumulation. At each time point, we observed a much higher expression level for the TRE genes than for TPS (Figure D in S1 File): this strong trehalase activity in *A*. *vaga* may explain why previous studies could not detect trehalose [34,36]. Moreover, we found the expression of *AvTpsA-A’* and *AvTreA-A’* to be significantly upregulated during drying process, reaching a peak at 37 h (when bdelloids had just entered into desiccated state) then decreasing to the control level during rehydration (Fig 5A and 5B). *AvTreB-B’* were significantly up-regulated in both drying and rehydration compared with the control (Fig 5D), indicating that those gene copies may be constitutively expressed with an upregulation at desiccation. In any case, the low level of transcription observed for the TPS genes of *A*. *vaga* and their expression at very specific time points may explain why previous studies failed to detect trehalose or the expression of the genes involved it its biosynthesis. Interestingly, this biological pattern is very similar to the trehalose metabolism/catabolism pathway observed in plants, where the trehalase activity is so high that overexpression of TPS genes does not result in an increase in trehalose unless trehalase activity is disrupted [31,74].

### The absence of trehalose phosphatase in bdelloid rotifers

No fusion of TPP domains with our TPS *A*. *vaga* genes was observed. For *AvTpsA-A’*, we hypothesize that the absence of TPP domains could be linked to their non-metazoan origin: indeed, the plant Class I TPS genes to which they are related do not contain any TPP active site [31].

Neither trehalose-6-phosphate phosphatase nor conserved motifs of the TPP domain were detected in the genome of *A*. *vaga*. Plant, bacteria, fungi and yeast cells contain a wide array of nonspecific phosphatases that are able to dephosphorylate trehalose-6 phosphate (T6P), although with a lower efficiency than TPP [14,75–77]. Therefore, it is possible that in *A*. *vaga* the trehalose biosynthesis pathway relies on such nonspecific phosphatases instead of TPP.

In *E*. *coli*, TPS and TPP enzymes are encoded by distinct genes (OtsA and OtsB) [33,78] (Figure A in S1 File), while in almost all eukaryotes, the TPS proteins are fused to the TPP domains (Figure A in S1 File) [33,69]. This organization suggests that all the eukaryotic TPS and TPP fused proteins descend from a common ancestor [33]. Avonce et al. 2010 [71] recently demonstrated the existence of a natural TPS-TPP bifunctional enzyme in the bacterial species *Cytophaga hutchinsonii*. Phylogenetic analysis further suggested that this prokaryotic gene might be related to the eukaryotic TPS-TPP fused genes. Among the four predicted TPS protein sequences of *A*. *vaga*, only *AvTpsA-A’* was susceptible to contain putative TPP regions in their extended C-terminus but none of the conserved short motifs of the TPP-like domain were detected [33,68], while the other two sequences (Ψ*AvTpsB* and Ψ*AvTpsB’*) clearly did not include TPP domains. Previous research have shown that although TPP-like domains have lost their TPP catalytic activity, these domains may still be required for correct protein conformation and stability as in the early bi-functional fusion enzyme [69].

#### The plausible role of trehalose metabolism in bdelloid rotifers

Trehalose is synthesized in many anhydrobiotic organisms and was once hypothesized to be a universal molecule that preserves cell integrity during desiccation. Nowadays, low trehalose levels found in some desiccation resistant animals like tardigrades or monogonont eggs, and in some plants, suggest that trehalose does not only act as bioprotective molecule but could be used alternatively as a signaling molecule.

In this study, we identified a series of genes in *A*. *vaga* that encode enzymes involved in trehalose synthesis and hydrolysis, some of which are substantially expressed. Although neither trehalose-6-phosphate phosphatase (TPP) nor conserved motifs of the TPP domain have been identified in *A*. *vaga*, we cannot exclude the possibility that *A*. *vaga* might synthesize very low amounts of endogenous trehalose using unspecific phosphatases. Interestingly, several features of the *A*. *vaga* trehalose metabolism, acquired through HGT, are highly similar to those observed in plants, such as the lack of trehalose accumulation, the existence of multiple copies of TPS and TRE genes, and the higher expression levels of TRE compared with TPS genes. In plants, both T6P and trehalose act as signaling molecules that induce metabolic changes, such as the accumulation of storage carbohydrates. As for many signaling molecules, the rapid degradation of trehalose may therefore be required to prevent its accumulation from interfering with the regulation of plant metabolism [15,79]. Furthermore, most Class I and Class II TPS proteins in plants have been suspected to have lost their TPS and TPP enzymatic activities but can still be detected, suggesting that other selective pressures are responsible for their persistence such as their role in other plant carbohydrate-related pathways involved in the development, sugar sensing and abiotic stress response [15,69,71]. Moreover, the synthesis of the trehalose precursor, trehalose-6-phosphate (T6P), has been shown to play a signaling role in plant growth and development and in sugar metabolism (sugar sensing under stress tolerance) [80–82]. T6P is also known as a signaling molecule in the yeast species *Saccharomyces cerevisiae* [83]. Therefore, there is growing evidence that it is not trehalose itself but more likely one of its precursors (such as T6P), or perhaps an enzyme involved in its synthesis (such as TPS), that serves as a signaling molecule controlling certain metabolic pathways [82,84]. However, it was also noticed that despite his key role in plant metabolism, T6P is present in very small quantities [85]. Our discovery of multiple copies of low-expression trehalose synthesis genes and highly expressed trehalose degradation genes in *A*. *vaga* suggests that trehalose and/or trehalose-6-phosphate may fulfill a signaling role in bdelloid rotifers as was proposed in plants and fungi, from which bdelloids may have acquired the TPS genes. This signaling role hypothesis is supported by the overexpression of *AvTpsA-A’* in early desiccated *A*. *vaga*, suggesting that T6P is involved as a messenger during dehydration. However, it remains necessary to confirm these hypotheses by measuring directly the appropriate metabolites and/ or enzyme activities. Finally, a recent study suggested that plant TPS may have scaffolding functions or can be involved in protein complexes [86]. Moreover, TPS from *Magnaporthe oryzae* was described as a key protein involved in enzyme activation, control of metabolic flux and transcriptional regulation of pathogenic fungus [87]. Therefore, we cannot exclude that bdelloids TPS have both catalytic and non-catalytic functions involved in the desiccation process. The discovery of TPS expression in bdelloids during their desiccation invites us to re-investigate the apparent paradox of trehalose’s absence in these organisms.

## Supporting Information

S1 Dataset qPCR data and statistical tests (assumptions of normality and homoscedasticity) performed for TPS and TRE genes. (XLSX)

S1 File Figure A: The metabolic pathways of trehalose: 1. Five distinct trehalose synthesis pathways are present in eukaryotes, bacteria and archaea. 2. Three distinct trehalose hydrolytic pathways have been described. UDP-Glc, uridine diphosphate glucose; Glc-6P, glucose-6-phosphate; T6P, trehalose-6-phosphate; UDP, uridine diphosphate; Glc-1P, glucose-1-phosphate; Glc, glucose; ADP, adenosine triphosphate (modified from [88]). Figure B: Expression study of the trehalose-6-phosphate synthase (TPS) and trehalase (TRE) genes in the bdelloid rotifer *Adineta vaga* during desiccation and rehydration: the six different time points included in the qPCR analyses are given. The cDNA libraries (in triplicates) were performed at the time points marked with “*”. Figure C: Protein sequence alignment of *E*. *coli* OtsA with the four *Adineta vaga* TPS domains, *A*. *thaliana* TPS1 (GI:15218422) and *P*. *brassicea* TPS1 (GI:160332824). Residues important for G6P and UDP binding in *E*. *coli* OtsA are indicated in red or green, respectively. Mutation compared with the OtsA model are shown in dark red and dark green for residues involved in binding G6P or UDP respectively. Figure D: Quantitative expression of the trehalose-6-phosphate synthase (TPS) and trehalase (TRE) genes in *A*. *vaga* submitted to drying and rehydration (based on triplicate RNAseq librairies). Error bars represent standard deviations. *ΨAvTpsB* and *ΨAvTpsB’*were not represented as there were no corresponding reads in the librairies. Figure E: Alignment (using DNASTAR) of degenerate (a) forward and (b) reverse primers with the four trehalose-6-phosphate genes of *A*. *vaga*.
(PDF)

S2 File Table A: Primers used for amplifying the trehalose-6-phosphate synthase (TPS) and trehalase (TRE) genes of *Adineta vaga* using PCR. Table B: Reference for sequences used in phylogeny related to trehalose-6-phosphate synthase (TPS) and trehalase (TRE) genes. Table C: Primers used for amplifying the trehalose-6-phosphate synthase (TPS), trehalase (TRE) and L40 genes of *A*. *vaga* using qPCR. Table D: Nucleotidic and proteic similarities between *A*. *vaga* TRE sequences. The numbers in each cell represent respectively the nucleotidic and proteic similarity percentages.
(PDF)
