# Supplementary material for: Against All Odds: Trehalose-6-Phosphate Synthase and Trehalase Genes in the Bdelloid Rotifer Adineta vaga Were Acquired by Horizontal Gene Transfer and Are Upregulated during Desiccation
Source: PLoS One. 2015 Jul 10;10(7):e0131313. doi: 10.1371/journal.pone.0131313 (PMC4498783; doi:10.1371/journal.pone.0131313)
Supplement: S1 File — Five distinct trehalose synthesis pathways are present in eukaryotes, bacteria and archaea. 2. Three distinct trehalose hydrolytic pathways have been described. UDP-Glc, uridine diphosphate glucose; Glc-6P, glucose-6-phosphate; T6P, trehalose-6-phosphate; UDP, uridine diphosphate; Glc-1P, glucose-1-phosphate; Glc, glucose; ADP, adenosine triphosphate (modified from [88]). Figure B: Expression study of the trehalose-6-phosphate synthase (TPS) and trehalase (TRE) genes in the bdelloid rotifer Adineta vaga during desiccation and rehydration: the six different time points included in the qPCR analyses are given. The cDNA libraries (in triplicates) were performed at the time points marked with “*”. Figure C: Protein sequence alignment of E. coli OtsA with the four Adineta vaga TPS domains, A. thaliana TPS1 (GI:15218422) and P. brassicea TPS1 (GI:160332824). Residues important for G6P and UDP binding in E. coli OtsA are indicated in red or green, respectively. Mutation compared with the OtsA model are shown in dark red and dark green for residues involved in binding G6P or UDP respectively. Figure D: Quantitative expression of the trehalose-6-phosphate synthase (TPS) and trehalase (TRE) genes in A. vaga submitted to drying and rehydration (based on triplicate RNAseq librairies). Error bars represent standard deviations. ΨAvTpsB and ΨAvTpsB’were not represented as there were no corresponding reads in the librairies. Figure E: Alignment (using DNASTAR) of degenerate (a) forward and (b) reverse primers with the four trehalose-6-phosphate genes of A. vaga. (PDF) [file pone.0131313.s002.pdf]

Figure A

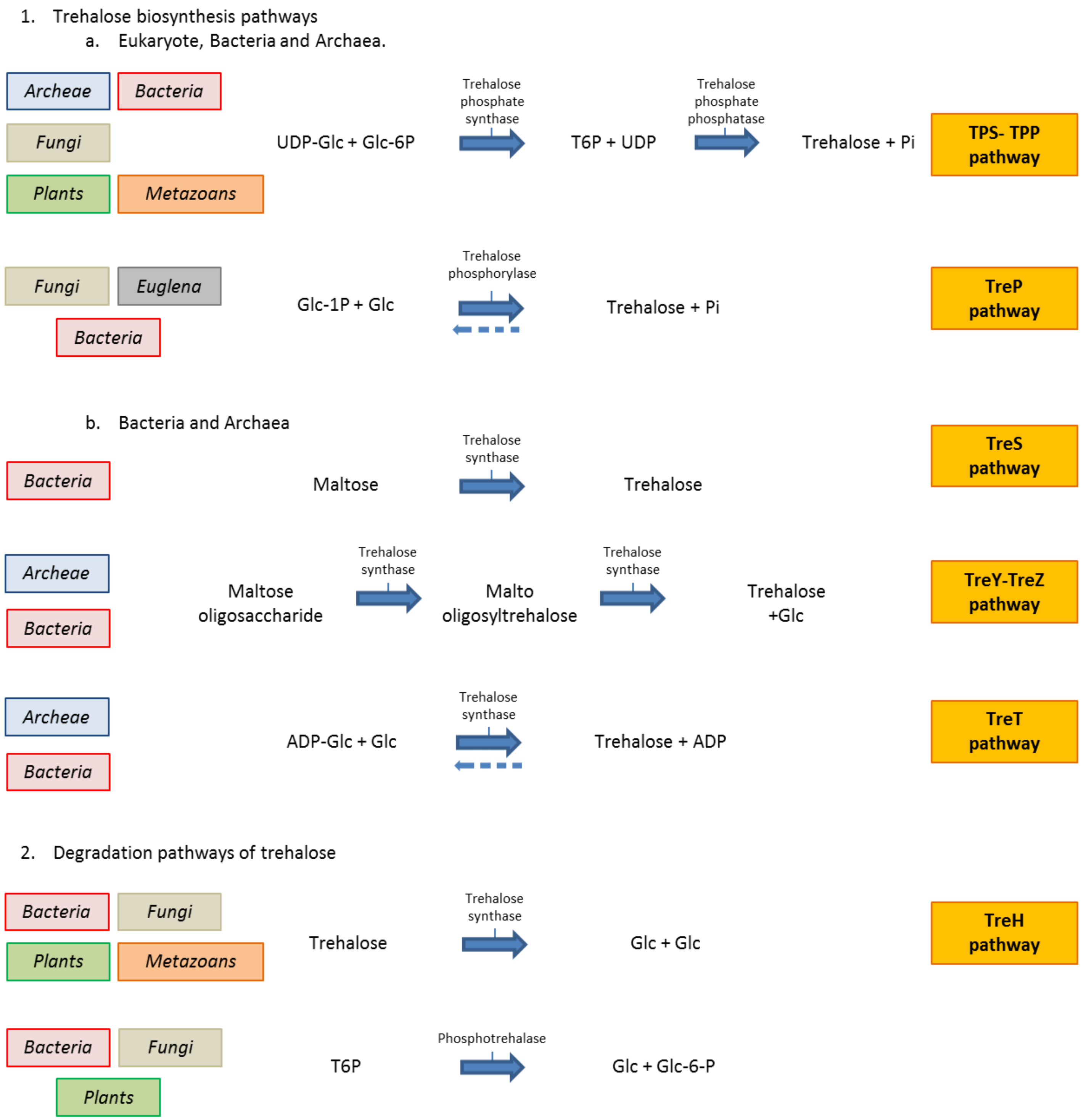
1. Trehalose biosynthesis pathways
a. Eukaryote, Bacteria and Archaea.
Archeae
Bacteria
Fungi
Plants
Metazoans
UDP-Glc + Glc-6P
Trehalose phosphate synthase
T6P + UDP
Trehalose phosphate phosphatase
Trehalose + Pi
TPS- TPP pathway
Fungi
Euglena
Bacteria
Glc-1P + Glc
Trehalose phosphorylase
Trehalose + Pi
TreP pathway
b. Bacteria and Archaea
Bacteria
Maltose
Trehalose synthase
Trehalose
TreS pathway
Archeae
Bacteria
Maltose oligosaccharide
Trehalose synthase
Malto oligosyltrehalose
Trehalose synthase
Trehalose +Glc
TreY-TreZ pathway
Archeae
Bacteria
ADP-Glc + Glc
Trehalose synthase
Trehalose + ADP
TreT pathway
2. Degradation pathways of trehalose
Bacteria
Fungi
Plants
Metazoans
Trehalose
Trehalose synthase
Glc + Glc
TreH pathway
Bacteria
Fungi
Plants
T6P
Phosphotrehalase
Glc + Glc-6-P

Figure B

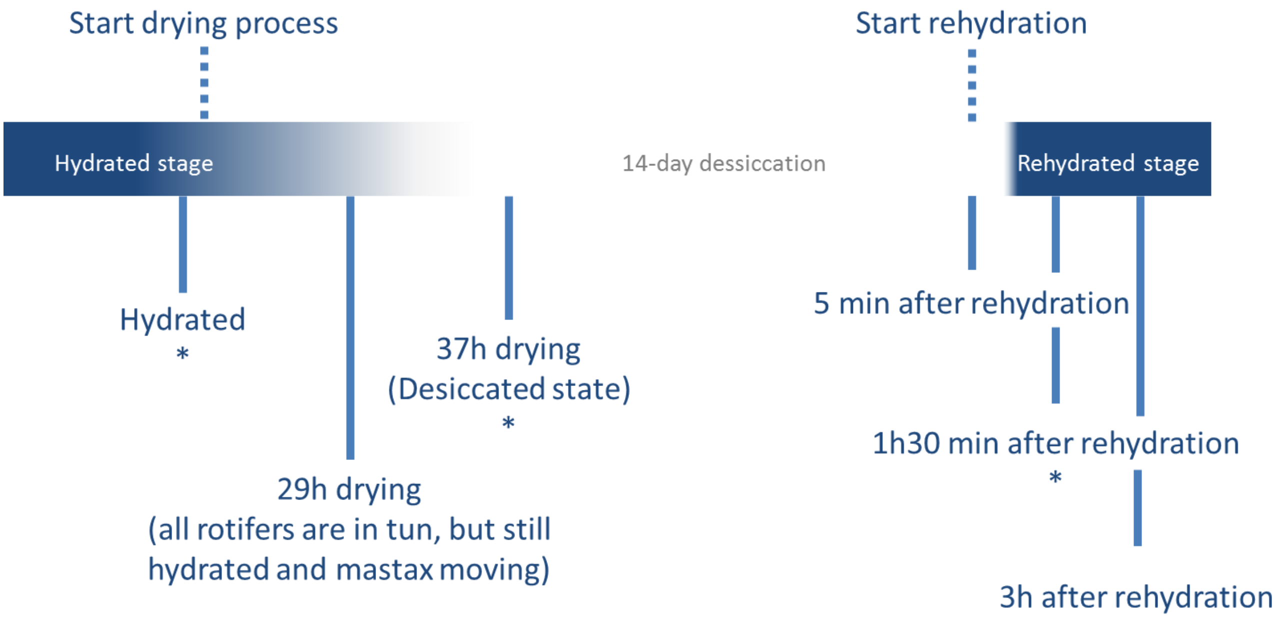
Start drying process
Hydrated stage
Hydrated
*
29h drying
(all rotifers are in tun, but still
hydrated and mastax moving)
37h drying
(Desiccated state)
*
14-day dessiccation
Start rehydration
Rehydrated stage
5 min after rehydration
1h30 min after rehydration
*
3h after rehydration

Figure C

```
                      ....|....| ....|....| ....|....| ....|....| ....|....|
                              5         15         25         35         45
E.coli OtsA           ---------- ---------- ---------- ---------- ----------
AvTpsA                MPLDT----- -----FLLPS SADIEQQEYQ LSMTSIPTHN HLIKDQKNI-
AvTpsA’               MPLDT----- -----FLLPS SADIGQQEYQ LSMTSIPTHN HLITDKKNI-
ΨAvTpsB               ---------- ---------- ---------- ---------- ----------
ΨAvTpsB’              ---------- ---------- ---------- ---------- ----------
A.thaliana TPS1       MPGNK--YNC SSSHIPLSRT ERLLRDRELR EKRKSNRARN PNDVAGSSEN
P.brassicae TPS1     MPDDREGFVD DDPRLMSLDL SSDDGDEFVR SEHREKVLQE RIVALQAQVT

                      ....|....| ....|....| ....|....| ....|....| ....|....|
                             55         65         75         85         95
E.coli OtsA           ---------- ---------- ---------- ---------- --MSRLVVVS
AvTpsA                ---------- ---------- ---------- ---------- --SARMIIVA
AvTpsA’               ---------- ---------- ---------- ---------- --SARMIIVA
ΨAvTpsB               ---------- ---------- ---------- ---------- --MGKIINVS
ΨAvTpsB’              ---------- ---------- ---------- ---------- --MGKIINVS
A.thaliana TPS1       SENDLRLEGD SSRQYVEQYL EGAAAAMAHD DACERQEVRP YNRQRLLVVA
P.brassicae TPS1     EERDLRRQ-- ARRQVAANRV ESPT------ ---------- --SFRLLVVA

                      ....|....| ....|....| ....|....| ....|....| ....|....|
                            105        115        125        135        145
E.coli OtsA           NRIAPPDE-- ---------- HAASAGGLAV GILGALKAAG GLWFGWSGET
AvTpsA                NRLPITMTIK SKRDGQSEVE FLSSSGGVAS ALTGISN--- -LWIGWPGGE
AvTpsA’               NRLPITMTIK SKRDGQSEVE FLSSSGGVAS ALTGISN--- -LWIGWPGGE
ΨAvTpsB               NRLPVTIA-- ---KSNHLYA FKPSSGGLKS CLESVRETVK FLWIGRPGLE
ΨAvTpsB’              NRLPVTIA-- ---KSNHLYT FKPSSRGLKS CLESVRETVQ FLWIGWPRLE
A.thaliana TPS1       NRLPVSAV-- --RRGEDSWS LEISAGGLVS ALLGVKE-FE ARWIGWAGVN
P.brassicae TPS1     NRLPMSLT-- --KDEFGQWS ATMSSGGLVS ALMGVKN-ME MKWIGWPGAT

                      ....|....| ....|....| ....|....| ....|....| ....|....|
                            155        165        175        185        195
E.coli OtsA           GNEDQPLKKV KKGNITWASF N--LSEQDLD EYYNQFSNAV LWPAFHYR--
AvTpsA                VRNAEDRKNV TEKLRKFDCI PVFLTAELCN LFYNGYCNDL LWPLLHYMPL
AvTpsA’               VRNAEDRKNV TEKLRKFDCI PVFLTAELCN LFYNGYCNDL LWPLLHYMPL
ΨAvTpsB               VDKSAEKQVG EELNENFECL PVYLMAELAN LYYEKYCNGT IWPILHGF--
ΨAvTpsB’              VDKSAEKQVE EESNENFECL PAYLTAELAN LYYEKYCNGT IWPILHEF--
A.thaliana TPS1       VPDEVGQKAL SKALAEKRCI PVFLDEEIVH QYYNGYCNNI LWPLFHYLGL
P.brassicae TPS1     IDNDADRQAI VTLLAEKNCK PVFLSKNLTD LYYSGYANNV IWPLFHYMPP

                      ....|....| ....|....| ....|....| ....|....| ....|....|
                            205        215        225        235        245
E.coli OtsA           -----LDLVQ FQRPAWDGYL RVNALLADKL LPLLQDDDII WIHDYHLLPF
AvTpsA                P----IEAIK SHDKRYLAYQ DANAQFAKIV LDNYDDGDIV WIHDYHLMLV
AvTpsA’               P----IEAIK SHDKRYLAYQ DANAQFAKIV LDNYDDGDIV WIHDYHLMLV
ΨAvTpsB               -----ISESE YRETYFEAYV KANELFANKI LETIEDGDTI WIHDYHLMLL
ΨAvTpsB’              -----ISESE YRETYFEAYV KANELFANKI LETIEDGDTI WIHDYHLMLL
A.thaliana TPS1       PQEDRLATTR SFQSQFAAYK KANQMFADVV NEHYEEGDVV WCHDYHLMFL
P.brassicae TPS1     P----IDHLK NSNLMFEAYE TVNQMFADEV LSEYRHGDIV WVHDYHLMLL

                      ....|....| ....|....| ....|....| ....|....| ....|....|
                            255        265        275        285        295
```

```
E.coli OtsA        AHELR-KRGV NNRIGFFLHI PFPTPEIFNA LPTYDTLLEQ LCDYDLLGFQ
AvTpsA             PSLLR-RAKP QMKIGFFFHT PFPCGEVYCT LPSRSELLLG VLNSNLIGFH
AvTpsA'            PSLLR-RAKP QMKIGFFFHT PFPCGEVYCT LPSRSELLLG VLNSNLIGFH
ΨAvTpsB            PKLIRMKCRK SIKIAFFLHI CWPYHETFRM VPHWEQICDS LLQADWVGFH
ΨAvTpsB'           PKLIRMKCRK SIKTAFFLHM CWPYHETFRM VPHWEQIRDS LLQADLVEFH
A.thaliana TPS1    PKCLK-EYNS KMKVGWFLHT PFPSSEIHRT LPSRSELLRS VLAADLVGFH
P.brassicae TPS1   PGILR-TASP KMQIGFFLHT PFPSSEIYRI LPQRDEIVHS LLSSDLIGFQ

                   ....|....| ....|....| ....|....| ....|....| ....|....|
                       305        315        325        335        345
E.coli OtsA        TENDRLAFLD CLSNLTRVTT RSAKSHTAWG KAFRTEVYPI GIEPKEIAKQ
AvTpsA             TDQYLRHFQS AVSRLLNVEY DNNQIRMQ-D IMSRLGVYPI GIDARKFVDT
AvTpsA'            TDQYLRHFQS AVSRLLNVEY DNNQIRMQ-D IMSRLGVYPI GIDARKFVDT
ΨAvTpsB            TLKYKKNFLN TCYELLNIK- PLDIIHHK-G RMVQIRNSIF SIQPKIIYNS
ΨAvTpsB'           TLKDKKNFLN TCYELLNIK- PLDIIHRK-G RMVQIRNSIF SIQPKTIYNS
A.thaliana TPS1    TYDYARHFVS ACTRILGLEG TPEGVEDQ-G RLTRVAAFPI GIDSDRFIRA
P.brassicae TPS1   VYDYCRHFMT SCTRVLGMDN PPNGGQLG-K GLATVGAFPI GIDPTRFTSA

                   ....|....| ....|....| ....|....| ....|....| ....|....|
                       355        365        375        385        395
E.coli OtsA        AAGP-LPPKL AQLKAELKNV QNIFSVERLD YSKGLPERFL AYEALLEKYP
AvTpsA             VASDSCQEYV NELKLHFAGR KVLLGIDRLD YIKGILQKML ALELFFENHP
AvTpsA'            VASDSCQDYV NELKLHFAGR KVLLGIDRLD YIKGILQKML ALELFFENHP
ΨAvTpsB            LETDRTKYEM NRIAEKYNGK KLIIGVDRID YIKGLDLKFK AYDIFLENSD
ΨAvTpsB'           LETDRTKYEM NRIAEKYKGK KLIIGFDRID CIKGLDLKFK VYDIFLENSD
A.thaliana TPS1    LEVPEVIQHM KELKERFAGR KVMLGVDRLD MIKGIPQKIL AFEKFLEENA
P.brassicae TPS1   VGTPRVIQYI DEFKKQFNSL TVIVGIDRLD PIKGILHKIH AMEQFFSDNP

                   ....|....| ....|....| ....|....| ....|....| ....|....|
                       405        415        425        435        445
E.coli OtsA        QHHGKIRYTQ IAPTSRGDVQ AYQDIRHQLE NEAGRINGKY GQLGWTPLYY
AvTpsA             EWIGKVILVQ VAVPSRTDVL EYQMFKAETH KLVGRINGRF GSVANVPICY
AvTpsA'            EWIGKVILVQ VAVPSRTDVL EYQMFKAETH KLVGRINGRF GSVANVPICY
ΨAvTpsB            N---NVIFEQ IAVPSRENIS IYKDYESRVA SLCTSLNEKH GRV----IEY
ΨAvTpsB'           N---NVIFEQ IAVPSRENIS IYKDYESRVA SLCTSLNEKH GRV----IEY
A.thaliana TPS1    NWRDKVVLLQ IAVPTRTDVP EYQKLTSQVH EIVGRINGRF GTLTAVPIHH
P.brassicae TPS1   NWVGKVVLVQ IAVPSRCDVP EYQRLAKETH EHVGRVNGRF GSISHAPIHF

                   ....|....| ....|....| ....|....| ....|....| ....|....|
                       455        465        475        485        495
E.coli OtsA        LNQHFDRKLL MKIFRYSDVG LVTPLRDGMN LVAKEYVAAQ DPAN-PGVLV
AvTpsA             LDQSMPFEKM CALYHCADVM MVTSIRDGMN LVSYEYIACQ KDST--GVLI
AvTpsA'            LDQSMPFEKM CALYHCADVM MVTSIRDGMN LVSYEYIACQ KDST--GVLI
ΨAvTpsB            RHESVDFHYL VALYRSADIC IVSSLADGLN LVAFEYIAAQ KDHS--GVLL
ΨAvTpsB'           RHESVDFHYL VALYRSADIC IVSLLADGLN LVAFEYIAAQ KDHS--GVLL
A.thaliana TPS1    LDRSLDFHAL CALYAVTDVA LVTSLRDGMN LVSYEFVACQ EAKK--GVLI
P.brassicae TPS1   LDQSIEFEKM CALYALADVC LITSLRDGMN LVSFEYVACQ TAMQGEGVLL

                   ....|....| ....|....| ....|....| ....|....| ....|....|
                       505        515        525        535        545
E.coli OtsA        LSQFAGAANE LTS-ALIVNP YDRDEVAAAL DRALTMSLAE RISRHAEMLD
AvTpsA             LSEFCGAAEL LDGGVLQINP WNINDVANAI YKALTMTENE RRKLSDIACN
AvTpsA'            LSEFCGAAEL LDGGVLQINP WNINDVANAI YKALTMSENE RRKLSDIACN
ΨAvTpsB            LSKFAGCSSI FRT-PMQFNP VNCDELAHLI KKAFEMSEVE RRTIQKNLFN
```

```
ΨAvTpsB'          LSKFAGCSSI FKT-PMQFNP VNCDELAHLI KKAFEMSEVE RRTIQKNLFN
A.thaliana TPS1   LSEFAGAAQS LGAGAILVNP WNITEVAASI GQALNMTAEE REKRHRHNFH
P.brassicae TPS1  LSEFCGASQS LGAGSIRINP WDVIETSKAI LYALEMSPAE RKEYHRYALN

                  ....|....| ....|....| ....|....| ....|....| ....|....|
                      555        565        575        585        595
E.coli OtsA       VIVKNDINHW QECFISDLKQ IVPRS----- ---AESQQRD KVATFPKLA-
AvTpsA            YVMGHTAKNW ATKFYEKLKD QEFVS-TNVS QPIHRNVSRD INEKISKSLN
AvTpsA'           YVMGHTAKNW ATKFYEKLKH QEFVP-TNVS QPIHRNASRD INEKISKSLN
ΨAvTpsB           TIINNTAEHW AKTLLKEMNF T--------- ---------- ----------
ΨAvTpsB'          TIINNTSEHW AKTLLKEMNF T--------- ---------- ----------
A.thaliana TPS1   HVKTHTAQEW AETFVSELND TVIEAQLRIS KVPPELPQHD AIQRYSKSNN
P.brassicae TPS1  YVQSHSAQHW ADSFVSKLRH CKKDV-VQVP VVPRRLDMDD VIEAFQKSSN

                  ....|....| ....|....| ....|....| ....|....| ....|....|
                      605        615        625        635        645
E.coli OtsA       ---------- ---------- ---------- ---------- ----------
AvTpsA            ---------- ---------- ---------- -------MEM TLSKFNSDQR
AvTpsA'           ---------- ---------- ---------- -------MEM TLSKFNSDQR
ΨAvTpsB           ---------- ---------- ---------- ---------- ----------
ΨAvTpsB'          ---------- ---------- ---------- ---------- ----------
A.thaliana TPS1   --RLLILGFN ATLTEPVDNQ GRRGDQIKEM DLNLHPELKG PLKALCSDPS
P.brassicae TPS1  CHRVLVLGLV GTLIQRRAS- GMPYDRFQMF AKCSAATLKA -LQKLSNDPQ

                  ....|....| ....|....| ....|....| ....|....| ....|....|
                      655        665        675        685        695
E.coli OtsA       ---------- ---------- ---------- ---------- ----------
AvTpsA            ELLIT----- -SLVD----- ---------- ---------- ----------
AvTpsA'           ELLIT----- -SLAD----- ---------- ---------- ----------
ΨAvTpsB           ---------- ---------- ---------- ---------- ----------
ΨAvTpsB'          ---------- ---------- ---------- ---------- ----------
A.thaliana TPS1   TTIVVLSGSS RSVLDKNFGE YDMWLAAENG MFLRLTNG-E WMTTMPEHLN
P.brassicae TPS1  TTVVVLTSRN RSLCDAVLGN NPVWVGAENG IFLKRGSGAE W-ENLQETVD

                  ....|....| ....|....| ....|....| ....|....| ....|....|
                      705        715        725        735        745
E.coli OtsA       ---------- ---------- ---------- ---------- ----------
AvTpsA            ------LIQR VEALSPTTID DTNEKDTCIS RSQYQDVGEL WG--------
AvTpsA'           ------LIHR VEALSPTTID DTNEKDTCIS RSQYQDVGEL WG--------
ΨAvTpsB           ---------- ---------- ---------- ---------- ----------
ΨAvTpsB'          ---------- ---------- ---------- ---------- ----------
A.thaliana TPS1   MEWVDSVKHV FKYFTERTPR SHFETRDTSL IWNYKYADIE FGRLQARDLL
P.brassicae TPS1  LSWTDEVLKV FNYFTERTPK SFVEKESTFI SWHYRDCDRE FGELQARDLL

                  ....|....| ....|....| ....|....| ....|....| ....|....|
                      755        765        775        785        795
E.coli OtsA       ---------- ---------- ---------- ---------- ----------
AvTpsA            ---W------ ---------- ----RKSSAT NSDTSNKVSS VLDSCESIHK
AvTpsA'           ---W------ ---------- ----RKSSAT NSDTSNKVSS VLDSCESIHK
ΨAvTpsB           ---------- ---------- ---------- ---------- ----------
ΨAvTpsB'          ---------- ---------- ---------- ---------- ----------
A.thaliana TPS1   QHLWTGPISN ASVDVVQGSR SVEVRAVGVT KGAAIDRILG EIVHSKSMTT
P.brassicae TPS1  MHLVAGPLVN TSTEVVHSSK LIQVRPAGVS KGNMADRIIS LIQQEHP---
```

```
                     ....|....| ....|....| ....|....| ....|....| ....|....|
                        805        815        825        835        845
E.coli OtsA          ---------- ---------- ---------- ---------- ----------
AvTpsA               NIATLEKTLS DKNNNDECFT VD-------- ---------- ----------
AvTpsA'              NIATLEKTLS DKNNNDECFT VD-------- ---------- ----------
ΨAvTpsB              ---------- ---------- ---------- ---------- ----------
ΨAvTpsB'             ---------- ---------- ---------- ---------- ----------
A.thaliana TPS1      PIDYVLCIGH FLGKDEDVYT FFEPELPSDM PAIARSRPSS DSGAKSSSGD
P.brassicae TPS1     NIGFLACLGD FLNRDEDLFQ HI-------- ---------- ----------

                     ....|....| ....|....| ....|....| ....|....| ....|....|
                        855        865        875        885        895
E.coli OtsA          ---------- ---------- ---------- ---------- ----------
AvTpsA               ---------- ---------- ---------- ---------- ----------
AvTpsA'              ---------- ---------- ---------- ---------- ----------
ΨAvTpsB              ---------- ---------- ---------- ---------- ----------
ΨAvTpsB'             ---------- ---------- ---------- ---------- ----------
A.thaliana TPS1      RRPPSKSTHN NNKSGSKSSS SSNSNNNNKS SQRSLQSERK SGSNHSLGNS
P.brassicae TPS1     ---------- ---AGSTSTT P--------- --------RR SASSLCL---

                     ....|....| ....|....| ....|....| ....|....| ....|....|
                        905        915        925        935        945
E.coli OtsA          ---------- ---------- ---------- ---------- ----------
AvTpsA               ---------- DNNNLSGKHL FL-------- ---------- ----------
AvTpsA'              ---------- DNNNLSGKYL FL-------- ---------- ----------
ΨAvTpsB              ---------- ---------- ---------- ---------- ----------
ΨAvTpsB'             ---------- ---------- ---------- ---------- ----------
A.thaliana TPS1      RRPSPEKISW NVLDLKGENY FSCAVGRTRT NARYLLGSPD DVVCFLEKLA
P.brassicae TPS1     ----PRSCYI ATINVGAKAG HAMKYVRSSV EVEQILGKLA DTIPNSSSAS

                     ....|....| ...
                        955
E.coli OtsA          ---------- ---
AvTpsA               ---------- ---
AvTpsA'              ---------- ---
ΨAvTpsB              ---------- ---
ΨAvTpsB'             ---------- ---
A.thaliana TPS1      DTTSSP---- ---
P.brassicae TPS1     SLSNGKNERR GXQ
```

Figure D

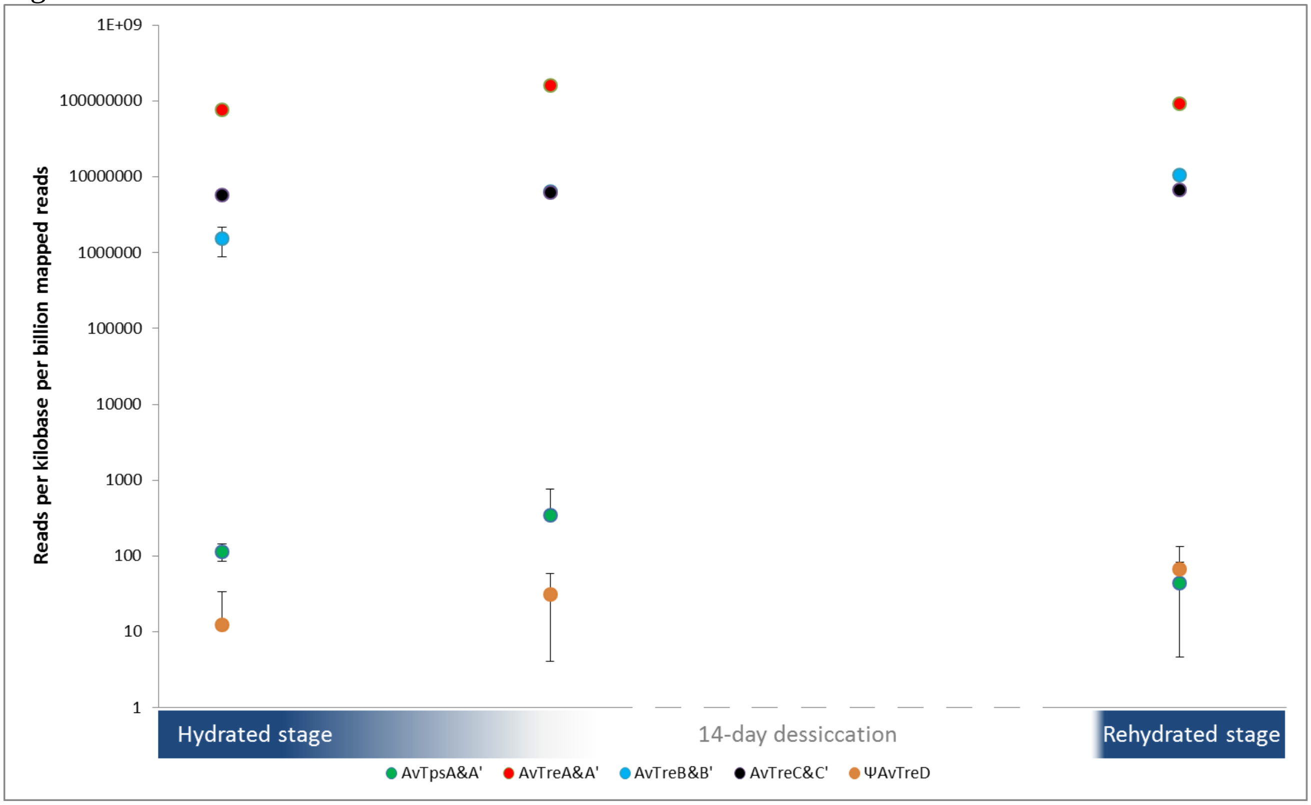
1E+09
100000000
10000000
1000000
100000
10000
1000
100
10
1
Reads per kilobase per billion mapped reads
Hydrated stage
14-day dessiccation
Rehydrated stage
AvTpsA&A'
AvTreA&A'
AvTreB&B'
AvTreC&C'
ΨAvTreD

Figure E

```
F_degenerate   -----------------------------------GGACTAGT--CAYGAYTAYCAYYTNATG
  AvTpsA'      AAGATTGTTTTGGATAATTACGATGATGGTGATATTGTTTGGATACATGATTATCATCTGATGTTAGTTCCA
  AvTpsA       AAGATTGTTTTGGATAATTACGATGATGGTGATATTGTTTGGATACATGATTATCATCTGATGTTAGTTCCA
  ΨAvTpsB      AATAAAATCTTAGAAACTATTGAAGATGGTGATACTATTTGGATACATGATTATCATCTAATGCTTCTACCA
  ΨAvTpsB'     AATAAAATCTTAGAAACTATTGAAGATGGCGATACTATTTGGATACATGATTATCATCTAATGCTTCTACCA

R_degenerate      ----------------------CATNARRTGRTARTCRTGACTAGTCC
  AvTpsA'         TTCAAAGCAGAAACTCATAAACTAGTTGGTCGTATCAATGGTCGATTCGGTAGTGTGG-CAAATGTTCCGAT
  AvTpsA          TTCAAAGCAGAAACTCATAAATTAGTTGGTCGTATCAATGGTCGATTCGGTAGTGTGG-CAAATGTTCCAAT
  ΨAvTpsB         TATGAATCACGAGTTGCATCTTTATGTACTTCCTTAAATGAAAAACATGGTCGAGTAATCGAATATCGACAT
  ΨAvTpsB'        TATGAATCACGAGTTGCATCTTTATGTACTTCCTTAAATGAAAAACATGGTCGAGTAATCGAATATCGACAT
```
