# Supplementary material for: Against All Odds: Trehalose-6-Phosphate Synthase and Trehalase Genes in the Bdelloid Rotifer Adineta vaga Were Acquired by Horizontal Gene Transfer and Are Upregulated during Desiccation
Source: PLoS One. 2015 Jul 10;10(7):e0131313. doi: 10.1371/journal.pone.0131313 (PMC4498783; doi:10.1371/journal.pone.0131313)
Supplement: S2 File — Table B: Reference for sequences used in phylogeny related to trehalose-6-phosphate synthase (TPS) and trehalase (TRE) genes. Table C: Primers used for amplifying the trehalose-6-phosphate synthase (TPS), trehalase (TRE) and L40 genes of A. vaga using qPCR. Table D: Nucleotidic and proteic similarities between A. vaga TRE sequences. The numbers in each cell represent respectively the nucleotidic and proteic similarity percentages. (PDF) [file pone.0131313.s003.pdf]

Table A

Gene	Primer sequence (5’->3’)	Annealing temp. (°C)
AvTpsA	5’-CGACCATTGATACGACCAACTAA-3’ and 5’-AAGTTTACTGTACCTTACCTTCACGT-3’	60
AvTpsA	5’-CCAATGGGATAGACACCCAG-3’ and 5’-GAAGATCGAAAAAATGTTACGGAA-3’	60
AvTpsA’	5’-CGACCATTGATACGACCAACTAG-3’ and 5’-AAGTTTACTGTACCTTACCTTCACGC-3’	60
AvTpsA’	5’-CCAATGGGATAGACACCCAA-3’ and 5’-GAAGATCGAAAAAATGTTACAGAG-3’	60
ΨAvTpsB	5’-AATGTTTTAGCCCAATGTTCAGC-3’ and 5’-ATCAAGCCGTTAGATATAATTCATCA-3’	60
ΨAvTpsB	5’-TCAGTTTCTAACGAATTGTAGATTATCTTC-3’ and 5’-TGTATGCATTTAAACCCAGTAGTG-3’	60
ΨAvTpsB’	5’-AATGTTTTAGCCCAATGTTCAGA-3’ and 5’-ATCAAACCGTTAGATATAATTCATCG-3’	60
ΨAvTpsB’	5’-TCAGTTTCTAATGAATTATAGATTGTCTTT-3’ and 5’-TGTATACATTTAAACCCAGCAGTC-3’	60
AvTreA	5’-AAAATGGACAATAGAAGAACATTGT-3’ and 5’-TCACGTAAAAACATACTTTCAACTTTC-3’	60
AvTreA’	5’-AAAATGGACAATAGAAGAACATTGC-3’ and 5’-ATCACGTAGAAACATACTTTCAACTTTT-3’	60
AvTreB	5’-ATCGGCAATTCGACAAAAAT-3’ and 5’-AATGATAAAACTTTCTTTTGACGTTTA-3’	60
AvTreB’	5’-ATCGGCAATTCGACAAAAAT-3’ and 5’-AATGATAAAACTTTCTTTTGACGTTTC-3’	60
AvTreC&C’	5’-AACTTGGACGATTGAAGAACATTGC-3’ and 5’-ATCATATAAAAATAATTTTTCAACCTGT-3’	60
ΨAvTreD	5’-ATTAGAAATTCAACAAAAATATTTACCG-3’ and 5’-AATGATACAATCTTTTTTTGACATTTT-3’	60

Table B

Species	GI/REF TPS	GI/ REF TREHALASE
Methanothermobacter_thermautotrophicus_MthTPS	GI:15679745	
Thermoplasma_acidophilum_TaTPS	GI:16082218	
Thermoplasma_volcanium	GI:13542085	
Bradyrhizobium_japonicum_BjTPS	GI:27375433	
Corynebacterium_diphtheriae_CdTPS	GI:38234530	
Corynebacterium_efficiens	GI:25029064	
Corynebacterium_glutamicum_CgTPS	GI:19553823	
Escherichia_coli_EcTPS	GI:16129848	
Mesorhizobium_loti_MlTPS	GI:13470877	
Mycobacterium_avium	GI:41406671	
Mycobacterium_bovis	GI:686002736	
Mycobacterium_leprae	GI:31794666	
Mycobacterium_tuberculosis_MtTPS	GI:15610626	
Ralstonia_solanacearum_RsTPS	GI:17549326	
Rhodopseudomonas_palustris	GI:39937718	
Salmonella_enterica	GI:16760877	
Streptomyces_avermitilis	GI:29830479	
Streptomyces_coelicolor	GI:21222683	
Synechococcus_sp.	GI:3152388	
Synechocystis_sp.	GI:16330944	
Xanthomonas_axonopodis_XaTPS	GI:21243937	
Xanthomonas_campestris	GI:21232512	
Ashbya_gossypii	GI:45187495	
Saccharomyces_cerevisiae_ScTPS1	GI:6319602	
Schizosaccharomyces_pombe_SpTPS1	GI:19115117	
Ashbya_gossypii_AgoTPS2	GI:45201228	
Ashbya_gossypii_AgoTPS3	GI:45190879	
Saccharomyces_cerevisiae_ScTPS2	GI:6320279	
Saccharomyces_cerevisiae_ScTPS3	GI:6323917	
Saccharomyces_cerevisiae_ScTSL1	GI:6323537	
Schizosaccharomyces_pombe_SpTPS2	GI:19115887	
Schizosaccharomyces_pombe_SpTPS3	GI:19115342	
Schizosaccharomyces_pombe_SpTPS4	GI:19114874	
Schizosaccharomyces_pombe_SpTPS5	GI:19115640	
Arabidopsis_thaliana_AtTPS1	GI:15218422	
Arabidopsis_thaliana_AtTPS2	GI:15219969	
Arabidopsis_thaliana_AtTPS3	GI:15219985	
Arabidopsis_thaliana_AtTPS4	GI:15234194	
Arabidopsis_thaliana_AtTPS5	GI:18414960	
Arabidopsis_thaliana_AtTPS6	GI:12324075	

Arabidopsis_thaliana_AtTPS7	GI:15221478
Arabidopsis_thaliana_AtTPS8	GI:30698024
Arabidopsis_thaliana_AtTPS9	GI:15220891
Arabidopsis_thaliana_AtTPS10	GI:15219002
Arabidopsis_thaliana_AtTPS11	GI:15224213
Oryza_sativa_OsTPS1	GI:34910846
Oryza_sativa_OsTPS2	GI:34902280
Oryza_sativa_OsTPS3	GI:34909526
Oryza_sativa_OsTPS4	GI:37806209
Oryza_sativa_OsTPS5	GI:42408334
Pyrobaculum_aerophilum_PaTPS1	GI:18312516
Caenorhabditis_elegans_TPS1	GI:32563851
Caenorhabditis_elegans_TPS2	GI:25147603
Caenorhabditis_remanei	GI:308480892
Trichinella_spiralis	GI:339242297
Aphelenchus_avenae	GI:74776556
Ascaris_suum	GI:541045445
Belgica_antarctica	GI:405132173
Polypedilum_vanderplanki	GI:225006178
Spodoptera_litura	GI:281372519
Apis_mellifera	GI:328776873
Drosophila_melanogaster	GI:19920676
Fenneropenaeus_chinensis	GI:189031345
Callinectes_sapidus	GI:218683226
Brachionus_calyciflorus	Unpublished David Mark Welch
Brachionus_manjavacas	Unpublished David Mark Welch
*Adineta_vaga_ΨAvTpsB**	GSADVT00043720001
*Adineta_vaga_ΨAvTpsB'**	GSADVT00063692001
Adineta_vaga_AvTpsA’	GSADVT00013173001
Adineta_vaga_AvTpsA	GSADVT00013141001
Batrachochytrium_dendrobatidis_BdTPS	GI:575471558
Cyphellophora_europaea	GI:568123472
Exophiala_dermatitidis_EdTPS	GI:378732233
Penicillium_roqueforti	GI:584410187
Aspergillus_fumigatus_AfTPS	GI:70981953
Pestalotiopsis_fici	GI:630012549
Plasmodiophora_brassicae_PbTPS1	GI:160332824
Plasmodiophora_brassicae_PbTPS2	GI:162138603
Physcomitrella_patens	GI:168034188
Micromonas_pusilla	GI:303273560
Triticum_aestivum_TaTPS	GI:206600899
Eucalyptus_grandis	GI:629104434
Morus_notabilis	GI:587864419

Rudanella_lutea	GI:518833291
Spirosoma_linguale	GI:502695096
Fibrella_aestuarina	GG505142564
Anditalea_andensis	GI:660634098
Adhaeribacter_aquaticus	GI:651341452
Dyadobacter_fermentans	GI:506292363
Pontibacter_sp.	GI:494928049
Fibrisoma_limi	GI:496582452
Spirosoma_linguale	GI:502694071
Flectobacillus_majorgi	GI:652618309
Cellvibrio_japonicus	GI:501462521
Emticicia_oligotrophica	GI:504842363
Solitalea_canadensis	GI:504492301
Eudoraea_adriatica	GI:648656801
Pedobacter_oryzae	GI:652504768
Leeuwenhoekiella_blandensis	GI:497464280
Fulvivirga_imtechensis	GI:497265757
Nafulsella_turpanensis	GI:648443684
Gramella_echinicola	GI:652540909
Erwinia_billingiae	GI:502965445
Pseudomonas_stutzeri	GI:489379966
Acidovorax_avenae	GI:503360766
Escherichia_coli	GI:446856967
Pseudomonas_aeruginosa	GI:499198074
Salmonella_enterica	GI:554967182
Harpegnathos_saltator	GI:307201597
Loa_loa	GI:393904141
Amphimedon_queenslandica	GI:340376019
Apolygus_lucorum	GI:486025413
Anolis_carolinensis	GI:637371485
Anisakis_simplex	GI:597915269
Bombus_terrestris	GI:340710210
Wuchereria_bancrofti	GI:402587875
Spodoptera_exigua	GI:156767499
Chelonia_mydas	GI:591375588
Polypedilum_vanderplanki	GI:225006187
Physcomitrella_patens	GI:134142850
Nicotiana_tabacum	GI:283131196
Glycine_max	GI:351723307
Aedes_aegypti	GI:157123786
Medicago_truncatula	GI:657376072
Homo_sapiens	GI:2789461
Artemia_franciscana	GI:285026271

Belgica_antarctica	GI:405132177
Ascaris_suum	GI:541045016
Arabidopsis_thaliana	GI:6651011
Theobroma_cacao	GI:590679553
Verticillium_dahliae	GI:346978146
Nostoc_punctiforme	GI:501380385
Adineta_vaga_AvTreA	GSADVT00056365001
Adineta_vaga_AvTreA'	GSADVT00056372001
Adineta_vaga_AvTreC-C'	GSADVT00001947001
Adineta_vaga_AvTreB	GSADVT00051563001
Adineta_vaga_AvTreB'	GSADVT00021696001
*Adineta_vaga_ΨAvTreD**	N/A
Brachionus_calyciflorus	Unpublished David Mark Welch
Drosophila_melanogaster	GI:17933716
Nicotiana_tabacum	GI:283131196
Medicago_truncatula	GI:116295207
Caenorhabditis_elegans	GI:32399460
Neurospora_crassa_NTH	GI:21622364
Laccaria_bicolor_NTH	GI:170098993
Schizosaccharomyces_pombe_NTH	GI:2909418
Neurospora_tetrasperma_NTH	GI:350294608
Aspergillus_nidulans_NTH	GI:2827392
Metarhizium_acridum_NTH	GI:45550221
Saccharomyces_cerevisiae_NTH	GI:151946395
Candida_albicans_NTH	GI:1246903
Laccaria_bicolor_ATH	GI:164649011
Metarhizium_anisopliae_ATH	GI:144227408
Spodoptera_exigua	GI:166236926
Saccharomyces_cerevisiae_ATH	GI:1061284
Mucilaginibacter_paludis	GI:495780604
Ceratitis_capitata	GI:498995050

Table C

Gene	Primer sequence (5'->3')	Annealing temp. (°C)
AvTpsA&A'	5'-TCTTCTCTGGCCACTTCTTCATT-3'and 5'-GGCAAGATATCGTTTATCATGTGATT-3'	60
AvTreA&A'	5'-AAGATTGGCCTAATTCCAAATG-3' and 5'-ATGGTGGTTGTGATCGTCCT-3'	60
AvTreB&B'	5'-TCCAGCTGATGAACCAACAC-3'and 5'-TTTCACGAAATCTTCCACCA-3'	60
AvTreC&C'	5'-CGAAGCTTTTCGGGAAGATA-3'and 5'-CCCATCCTGATTCTGCACTT-3'	60
L40	5'-TTGAAGTTGAACCATCCGATAC-3'and 5'-GTTGATCAGGTGGAATACCTTCTT-3'	60

Table D

	AvTreA	*AvTreA'*	*AvTreB*	*AvTreB'*	*AvTreC*	*AvTreC'*	*ΨAvTreD*
AvTreA							
AvTreA'	99,3-99,4						
AvTreB	58,4-42,7	58,5-42,8					
AvTreB'	58,7-42,4	58,8-42,2	97,6-98,3				
AvTreC	75,3-69,9	75,5-69,6	58,8-42,5	59-42,6			
AvTreC'	75,3-69,9	75,5-69,6	58,8-42,5	59-42,6	100-100		
ΨAvTreD	59,3-43	59,5-43	85,7-79,2	86-79,2	58-42,2	58-42,2	
